# Transition Metal Catalysis for the Asymmetric Synthesis of 2-Arylethylamines: A Review of the New Millennium

**DOI:** 10.3390/molecules30081721

**Published:** 2025-04-11

**Authors:** Alejandro Manchado, Ángel García-González, Carlos T. Nieto, Nieves G. Ledesma, David Díez, Narciso M. Garrido

**Affiliations:** Department of Organic Chemistry, Faculty of Chemical Sciences, University of Salamanca, Pl. Caídos, s/n, 37008 Salamanca, Spain; alex92mc@usal.es (A.M.); u149349@usal.es (Á.G.-G.); eneas@usal.es (C.T.N.); nievesgarcial11@usal.es (N.G.L.); ddm@usal.es (D.D.)

**Keywords:** 2-phenethylamine, 2-arylethylamine, asymmetric synthesis, transition metal catalysis, metal catalysis, photocatalysis, transition metal-catalyzed asymmetric catalysis and synthesis, kinetic resolution, bioactive compounds, molecular design, organic synthesis

## Abstract

The 2-arylethylamine motif is very well-known in medicinal chemistry because of its interesting properties when it comes to interacting with the Central Neural System thanks to its ability to pass the blood–brain barrier. This nitrogen-containing family of compounds is of great interest in synthetic organic chemistry and, when it comes to its asymmetric synthesis, great challenges can be faced in order to obtain the chiral purity required in the drug industry. Thus, we provide a concise transition metal review presenting the recent advances in the synthesis of chiral 2-arylethylamines using transition metals as the main catalysts in the introduction of chirality. Both conventional and photocatalysis methods will be covered, considering the main transition metal used in the studies.

## 1. Introduction

**Figure 1:**
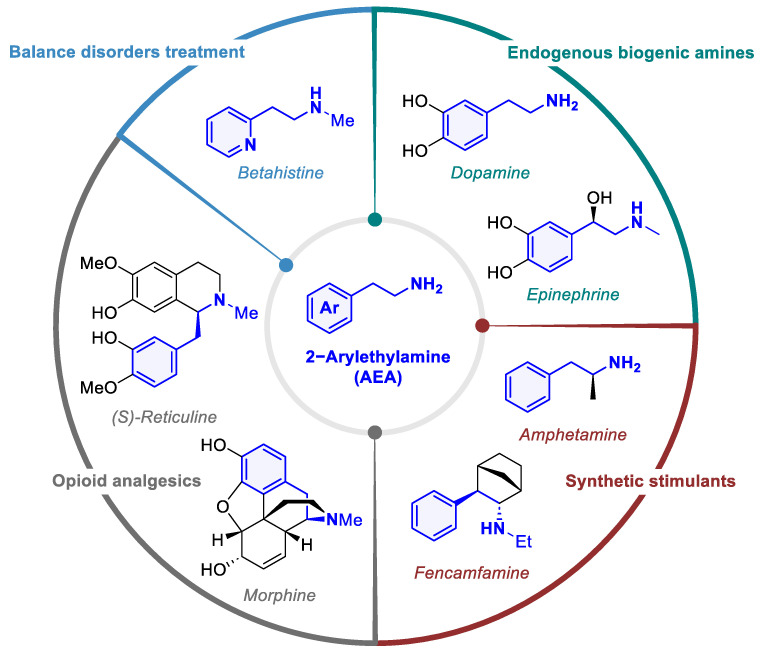
Representative examples of 2-arylethylamine derivatives.

Phenethylamine derivatives constitute a well-established class of bioactive compounds with significant pharmacological relevance [1,2]. Their relatively low molecular weight and amphiphilic nature facilitate their passage through the blood–brain barrier, allowing direct interaction with dopaminergic neurons and modulating key physiological processes such as motor control, stress response, and mood regulation [3]. These scaffolds, whether of natural origin (e.g., dopamine) or synthetically designed (e.g., amphetamine and its analogs), serve as the backbone for a wide range of central nervous system stimulants used in the treatment of neurological and psychiatric disorders (Figure 1).

Our research group has previously reviewed the medicinal chemistry of 2-phenethylamines and related scaffolds, examining key pharmacophores and their therapeutic relevance [4], as well as extending this analysis to bioactive 2-heteroarylethylamines [5]. More recently, we examined metal-free strategies for the asymmetric synthesis of 2-arylethylamines (AEA), covering chiral induction catalysis, organocatalysis, organophotocatalysis, and enzymatic approaches [6].

Previous reviews on this topic have discussed multiple aspects of the AEA syntheses, emphasizing different strategies and catalytic approaches. Some have focused on the advances made in asymmetric direct hydrogenation of C=N bonds with metal catalysts, such as those by Wencel-Delord et al. in 2021 [7] and Riera et al. in 2022 [8]. Other approaches have also been explored, such as enzymes and chemoenzymatic cascades making use of amine transaminases by Rueping et al. in 2023 [9], as well as the broader review by Moran et al. [10] encompassing both metal-catalyzed and metal-free synthetic methods towards β-(hetero)arylethylamines. From a substrate-based perspective, alkene functionalization has emerged as a powerful strategy for AEA synthesis, as highlighted in reviews by Wang et al. in 2022 [11] and Zhang et al. in 2025 [12]. Additionally, aziridines have been recently investigated by Lee et al. [13] as key intermediates for the synthesis of medicinally relevant chiral amines, while Xiaowei et al. reviewed the asymmetric photocatalytic synthesis of azaarene derivatives in 2022 [14]. Also, collectively, these studies examine the plethora of options available in the synthesis of AEAs, each providing some unique advantages and scope within the field.

The use of transition metal catalysis in the synthesis of enantioenriched compounds has been proved to be great tool in the synthesis of bioactive compounds which are suitable for the pharmaceutical industry [7,8,9]. In comparison with non-transition metal methodologies, this field has a large number of potential applications, both when it comes to suitable substrates and the moieties synthesized [6]. As for the synthesis of chiral AEAs, using transition metal catalysis has proved to be a path towards the development of interesting strategies, such as asymmetric hydrogenation of C=N bonds, asymmetric ring-openings, or C–N cross-coupling reactions, among others. These key methodologies, with which it is difficult to achieve success in their organocatalytic counterpart [6], are the main core of the synthetic advances in the synthesis of asymmetric 2-arylethylamine.

Due to the lack of reviews considering both methodology and substrate scopes, the current work aims to contribute to this body of knowledge by providing a comprehensive review of asymmetric metal catalysis for chiral AEA synthesis that, together with our previous metal-free review, completes the current domain of asymmetric synthesis of the aforementioned family of compounds in the new millennium.

**Figure 2:**
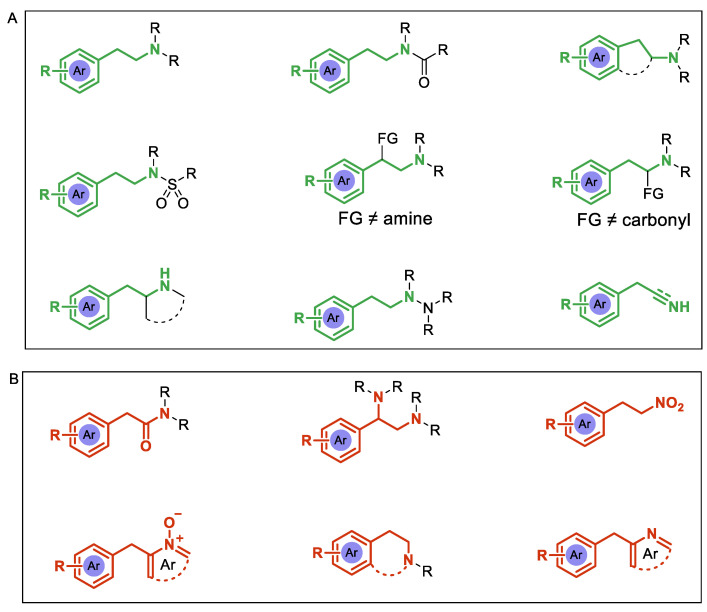
(**A**) 2-arylethylamines covered in this review. (**B**) Compounds not covered in this review.

Thus, we report the main advances in the last millennium in asymmetric synthesis of 2-AEA when using transition metal catalysis, covering the chiral synthesis of AEA skeletons considering different substituents, functional groups, and aryl/heteroaryl rings and their combinations, such as those shown in Figure 2A. Chirality is considered either in C-1, C-2, or in both carbon atoms. Also, different moieties in which nitrogen is two carbons apart from an aryl group are not covered, as shown in Figure 2B.

**Figure 3:**
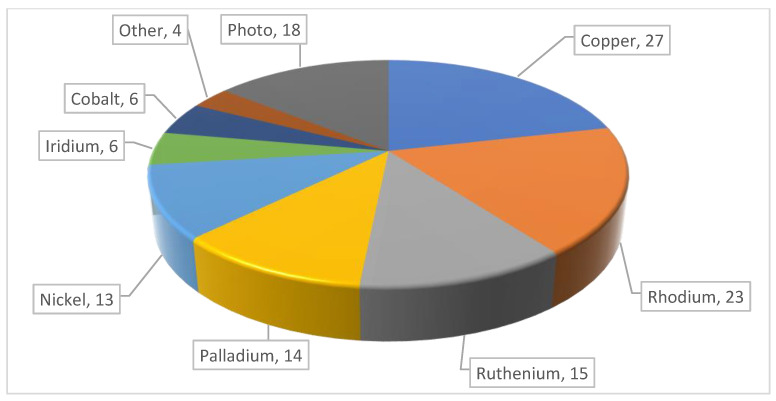
Number of studies on chiral AEA based on the transition metal used to perform the corresponding methodology, as well as photocatalysis.

This review is organized based on the transition metal used to enable the main step in the production of asymmetric AEA, with the transition metals organized based on the number of studies on the topic (Figure 3). Also, photocatalysis-based methodologies will be presented separately, and main and novel catalytic cycles will be presented and discussed.

## 2. Conventional Metal Catalysis

### 2.1. Copper

**Scheme 1:**
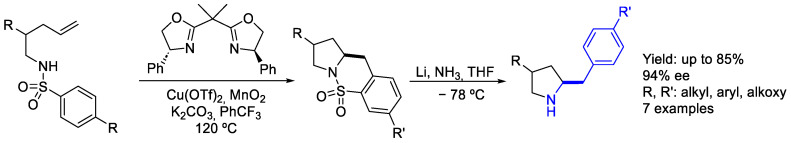
Chiral synthesis of AEA from sultam derivatives by Chemler et al. in 2017.

In 2007, Chemler et al. [15] reported a Cu(II)-catalyzed enantioselective intramolecular carboamination of alkenes, involving intramolecular addition of arylsulfonamides across terminal alkenes to provide chiral sultams. Chirality was enabled by a chiral copper–oxazolinyl complex and the corresponding products could be derivatized in order to obtain chiral AEA, in which the nitrogen atom is embedded into a pyrrolidine ring (Scheme 1).

**Scheme 2:**
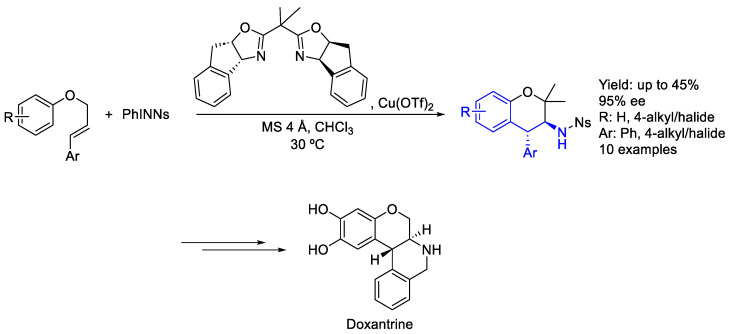
Chiral arylchorman-based AEA synthesized by Hajra et al. in 2011 with copper catalysis.

In 2011, Hajra et al. [16] reported a catalytic enantioselective one-pot aziridoarylation reaction of aryl cinnamyl ethers followed by an intramolecular arylation (Friedel–Crafts) reaction towards the synthesis of an interesting chiral AEA based on arylchromans. In situ-generated tethered aziridine provides easy access to *N*-sulfonyl-protected *trans*-3-amino-4-arylchromans in moderate yields and good ee by using the chiral organo-copper complex as a catalyst (Scheme 2).

**Scheme 3:**
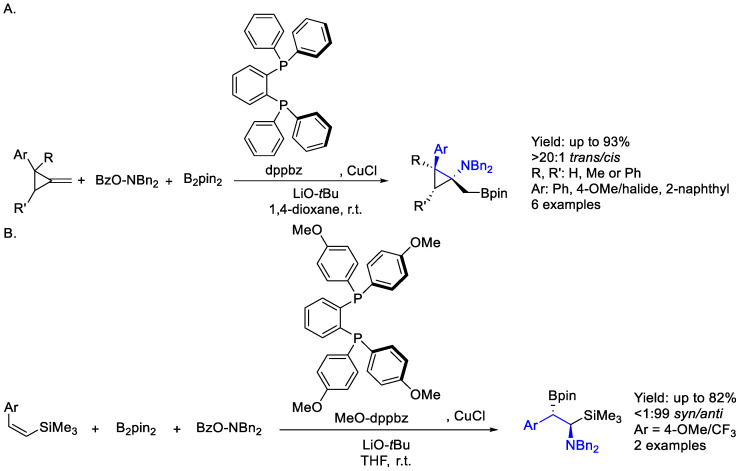
Cyclopropane-based chiral AEA (**A**) and β-boryl-α-aminosilane-based AEA (**B**) synthesized in 2014 and in 2016 by Miura et al., respectively.

Three years later, in 2014, Miura et al. [17] developed the asymmetric synthesis of (borylmethyl)cyclopropylamines by aminoboration of methylenectclopropanes using chiral-copper catalysis. This highly regio- and stereoselective process could afford interesting AEA in excellent yields and dr, which could be derivatized in order to obtain versatile *trans*-2-phenylcyclopropylamine derivatives, interesting potential moieties in medicinal chemistry (Scheme 3A). Two years later [18], they reported the synthesis of β-boryl-α-aminosilanes. This copper-catalyzed regioselective and stereospecific aminoboration of vinylsilanes with bis(pinacolato)diboron(pinB-Bpin) and hydroxylamines afforded asymmetric silyl boron AEA in good yields and dr (Scheme 3B).

**Scheme 4:**
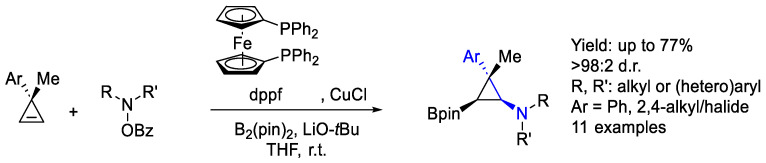
Asymmetric synthesis of AEA based on cyclopropylboronates by Tortosa et al. in 2014.

In 2014, Tortosa et al. [19] reported the first asymmetric synthesis of cyclopropylboronates with a quaternary stereocenter by copper-catalyzed diastereo- and enantioselective desymmetrization of cyclopropenes. Trapping the cyclopropylcopper intermediate with electrophilic amines allowed for the synthesis of chiral AEA with good yields and ee (Scheme 4).

**Scheme 5:**
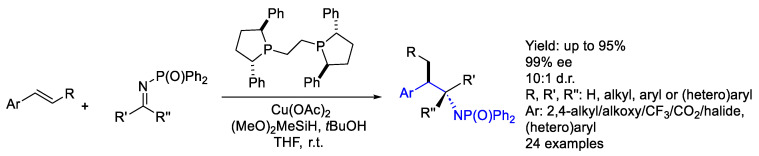
Chiral AEA by copper catalysis synthesized in 2016 by Buchwald et al.

Two years later, Buchwald et al. [20]. developed the enantioselective synthesis of an interesting AEA by copper catalysis. Starting from styrene-derived nucleophiles and imine derivatives, this intermolecular enantioselective addition of styrenes to imines afforded highly enantiomerically enriched amines bearing contiguous stereocenters in excellent yields and ee. This method relied on the use of styrenes as latent carbanion equivalents via the intermediacy of catalytically generated benzyl-copper derivatives. Interestingly, mechanistic studies remarked on the preferential styrene hydrocupration in the presence of an imine with the Ph-BPE-derived copper catalyst (Scheme 5).

**Scheme 6:**
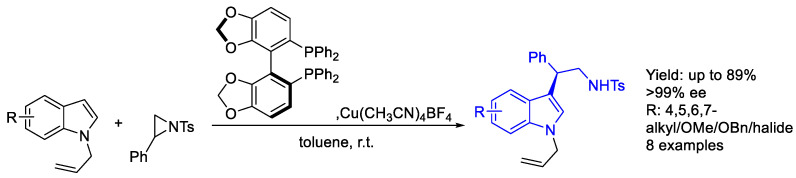
Asymmetric synthesis of indolethylamines by Jia et al. in 2016.

Also in 2016, Jia et al. [21] reported a Cu(I)-catalyzed enantioselective Friedel–Crafts alkylation of indoles with 2-aryl-*N*-sulfonylaziridines (Scheme 6). This process afforded indole-substituted chiral AEA in good yields and ee. Starting from racemic substituted aziridine, asymmetric addition by ring-opening reaction with indoles could be possible due to chiral Cu(I)/(*S*)-Segphos catalyst.

**Scheme 7:**
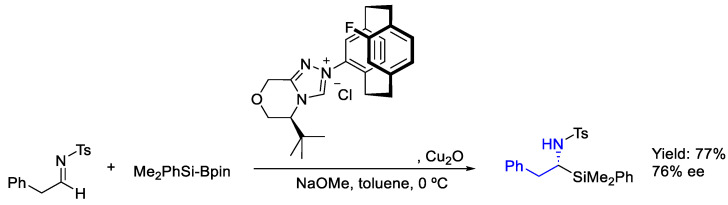
Chiral AEA synthesis via transannular organo-copper catalyst by Ma et al. in 2016.

Ma et al., also in 2016 [22], developed the asymmetric synthesis of a-silyl *N*-tosylamides by using [2.2]paracyclophane-based *N*-heterocyclic carbene in combination with a copper complex. The dual role of the new carbene precursor as an organocatalyst and as a ligand of the copper catalyst was crucial in the synthesis of the desired products. Starting from the corresponding imines, this methodology could afford a particular chiral α-silyl AEA in good yield and ee (Scheme 7), explained by the donor capacity of the carbene center through the transannular electronic effects, which significantly alters the activity of the resulting copper catalyst.

**Scheme 8:**
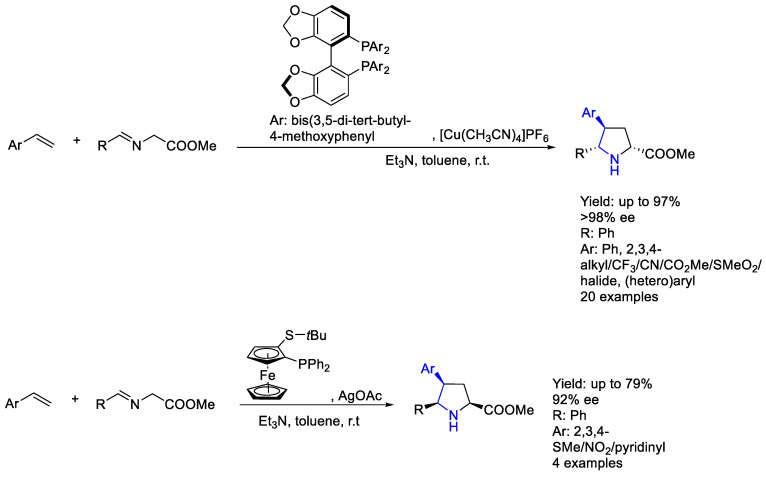
Asymmetric synthesis of pyrrolidine-based AEA by Carretero et al. in 2016.

In the same year, Carretero et al. [23] reported the direct synthesis of polisubstituted chiral pyrrolidines by catalytic asymmetric 1,3-dipolar cycloaddition reactions of azomethine ylides, enabled by chiral Cu(I)/(*R*)-DTBM-Segphos or Ag(I)/(*R*)-Fesuphos complexes. This methodology could allow total control of the stereocenters by using different copper or silver complexes, resulting in either *exo*- or *endo*-4-aryl substituted pyrrolidine with high diastereoselectivity and excellent enantioselectivity (Scheme 8).

**Scheme 9:**
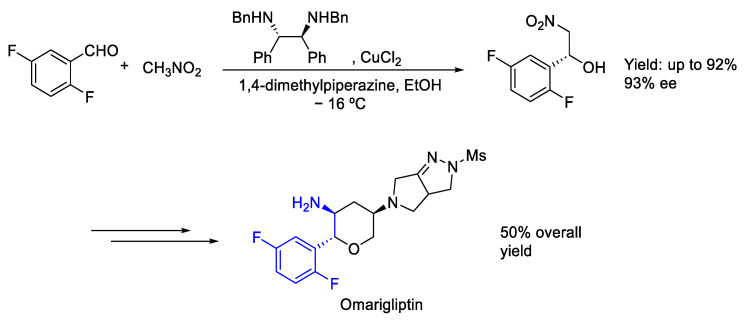
Omarigliptin synthesis in 2017 by Chung et al.

In 2017, Chung et al. [24] reported the formal synthesis of Omarigliptin, a long-acting DPP-4 inhibitor. The key step in the methodology was the asymmetric synthesis of a β-phenethylnitro compound from the corresponding benzaldehyde derivative and nitromethane, enabled by the chiral copper–dibenzylamine complex (Scheme 9). The subsequent treatment of the substrate led to chiral AEA Omarigliptin in excellent yield and ee.

**Scheme 10:**
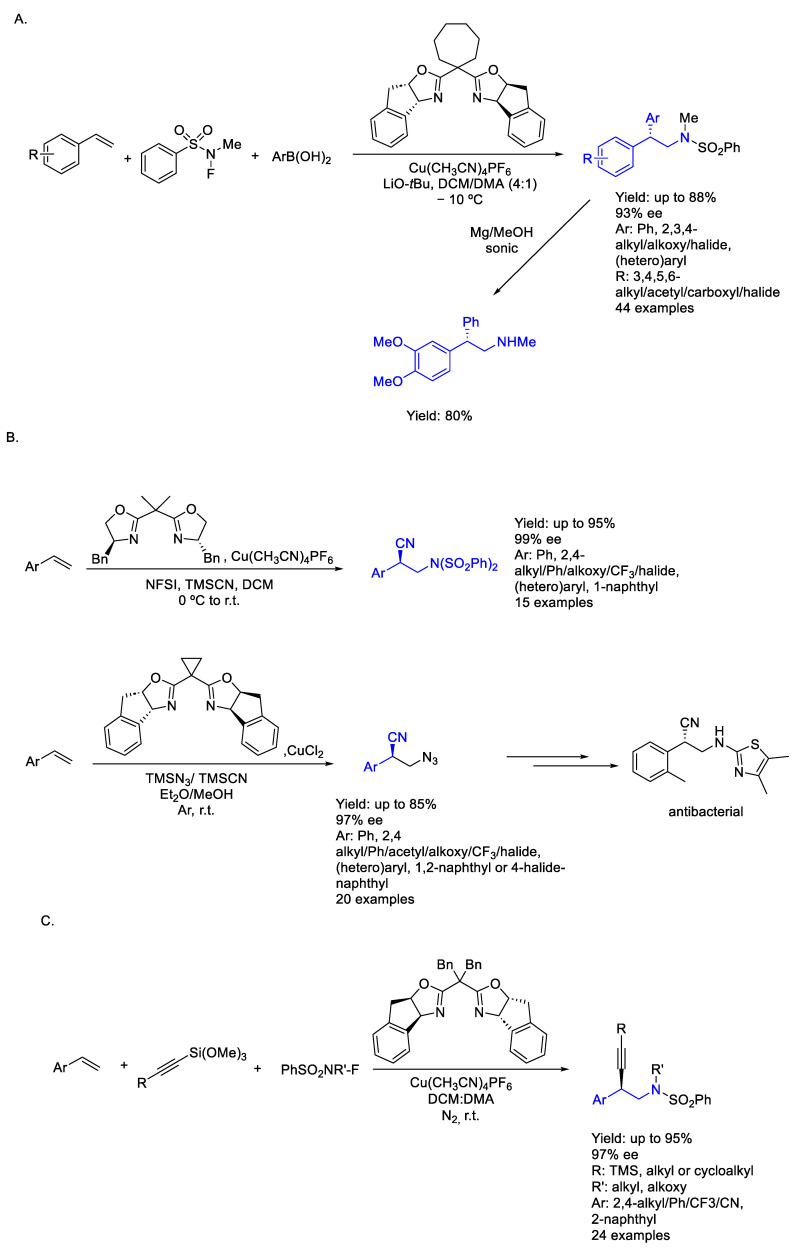
(**A**) *N*-sulfonyl-based AEA via copper catalysis by Liu et al. in 2017. (**B**) Amino- and azidocyanation towards the synthesis of an interesting AEA with high synthetic potential synthesized in 2017 by Liu et al. (**C**) Intermolecular aminoalkynylation towards the synthesis of an asymmetric AEA by Liu et al. in 2012.

Liu et al. have shown great success when it comes to the synthesis of chiral AEA using organo-copper complexes as transition metal catalysts. In 2017 [25], they reported the asymmetric copper-catalyzed intermolecular aminoarylation of styrenes. Starting from the corresponding styrene derivative and *N*-fluoro-*N*-alkylsulfonamide, several chiral AEA were synthesized in good yields and ee by its addition to styrene, so the generated benzylic radical could couple with a chiral L*Cu(II)Ar complex (Scheme 10A). Also, they reported the enantioselective copper-catalyzed intermolecular amino- and azidocyanation of styrenes in order to synthesize an interesting AEA in which the nitrogen functional group consisted of either a sulfonamide, a nitrile, or an azide [26]. By using organo-copper complexes, a variety of enantiomerically enriched β-amino/azido alkylnitriles were efficiently synthesized in excellent yields and ee. Also, the corresponding cyano-azido compounds could be derivatized in order to obtain bioactive compounds as antibacterials (Scheme 10B). Finally, in 2021 [27], following their studies in synthetic applications of styrene derivatives when it comes to organo-copper catalysis, they reported an enantioselective intermolecular aminoalkynylation using *N*-fluoro-*N*-alkylsulfonamides as nitrogen-centered radical precursors and alkynyltrimethoxysilanes as alkynylating reagents. This methodology tolerates wide substrate scope, high functional group tolerance, and mild conditions, and its products can be derivatized in order to obtain interesting synthons when it comes to both synthetic and medical applications (Scheme 10C).

**Scheme 11:**
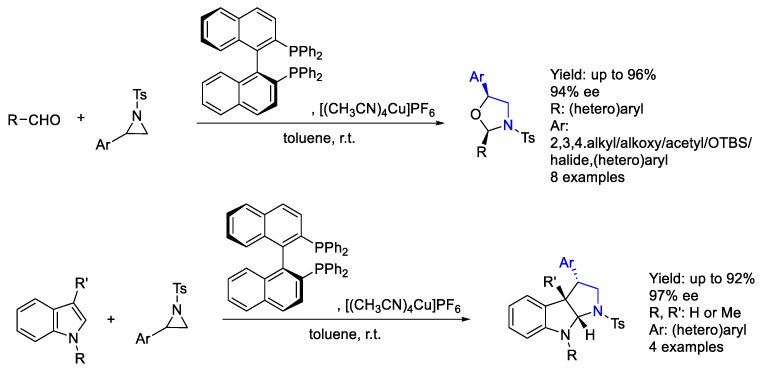
Oxazolidines and dihydroindolopyrrolidines synthesized by Chai et al. in 2018.

In 2018, Chai et al. [28] reported the synthesis of a chiral AEA by copper-catalyzed dynamic kinetic asymmetric transformation of racemic *N*-sulfonylaziridines. Interestingly, different products were afforded when starting from either aryl aldehyde derivatives or indol derivatives, via C–N bond cleavage with nucleophiles, including [3 + 2] annulations with (hetero)aromaticaldehydes and 1,3-disubstituted indoles, an asymmetric Friedel–Crafts-type reaction with electron-rich (hetero)arenes and asymmetric aminolysis with amines, resulting in a chiral AEA in which the nitrogen atom is embedded into an oxazolidine or into a fused dihydroindolopyrrolidine, respectively (Scheme 11). Thus, excellent yields and ee were achieved.

**Scheme 12:**
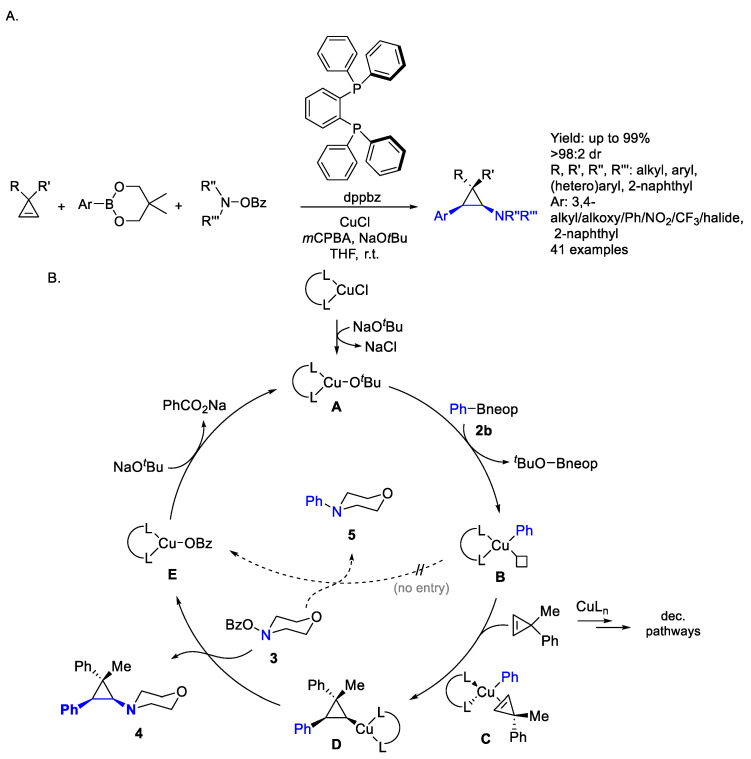
(**A**) Chiral AEA by Zhao et al. in 2019. (**B**) Proposed mechanism for the copper-catalyzed reaction.

In 2019, Zhao et al. [29]. developed the synthesis of chiral 2-arylcyclopropylamines enabled by copper-catalyzed cyclopropene carbometallation with an organo-boron reagent. This three-component synthesis afforded a polisubstituted AEA in excellent yields and ee. By using different chiral organic complexes, a large variety of compounds could be synthesized, representing the first example of highly enantioselective multicomponent cyclopropane synthesis (Scheme 12A). Mechanistic insights proposed that oxidative trapping of copper complex D by *O*-benzoyl hydroxylamine 3 delivered the desired products, while N insertion in early produced copper complex B did not occur, as shown in the proposed catalytic cycle (Scheme 12B).

**Scheme 13:**
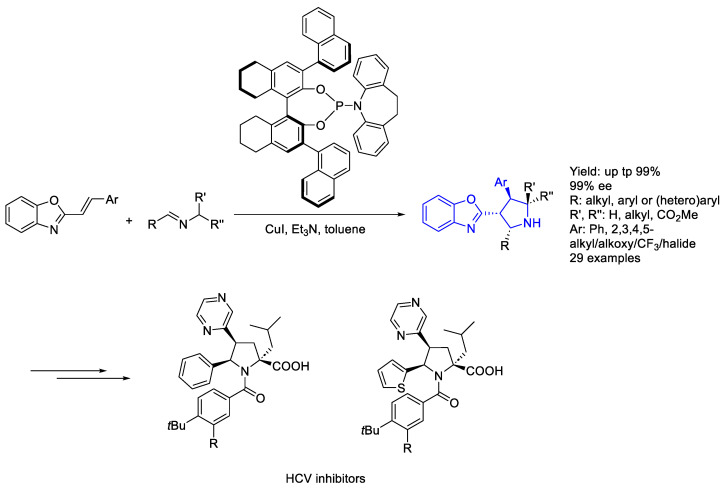
HCV derivatized from chiral AEA by Wang et al. in 2021.

In 2021, Wang et al. [30] developed the synthesis of an interesting polycyclic chiral AEA by Cu(I)-catalyzed asymmetric 1,3-dipolar cycloaddition of azomethine ylides in excellent yields and ee. The resulting asymmetric AEA consisted of pyrrolidine substrates and could be obtained in gram-scale, as well as derivatized in order to obtain key intermediates in the synthesis of bioactive compounds, such as HCV inhibitors. Various β-substituted alkenyl heteroarenes were successfully employed as dipolarophiles for the first time (Scheme 13). Also, DFT calculations proposed uncommon dual activation/coordination of both the dipole and dipolarophile substrates by the metal, in which a sterically bulky, rigid, and monodentate phosphoramidite ligand with triplehomoaxial chirality played a pivotal role in providing an effective chiral pocket around the metal center. The additional coordination of the heteroatom in the dipolarophile substrate to copper is also critical for the exclusive diastereoselectivity and enhanced reactivity.

**Scheme 14:**
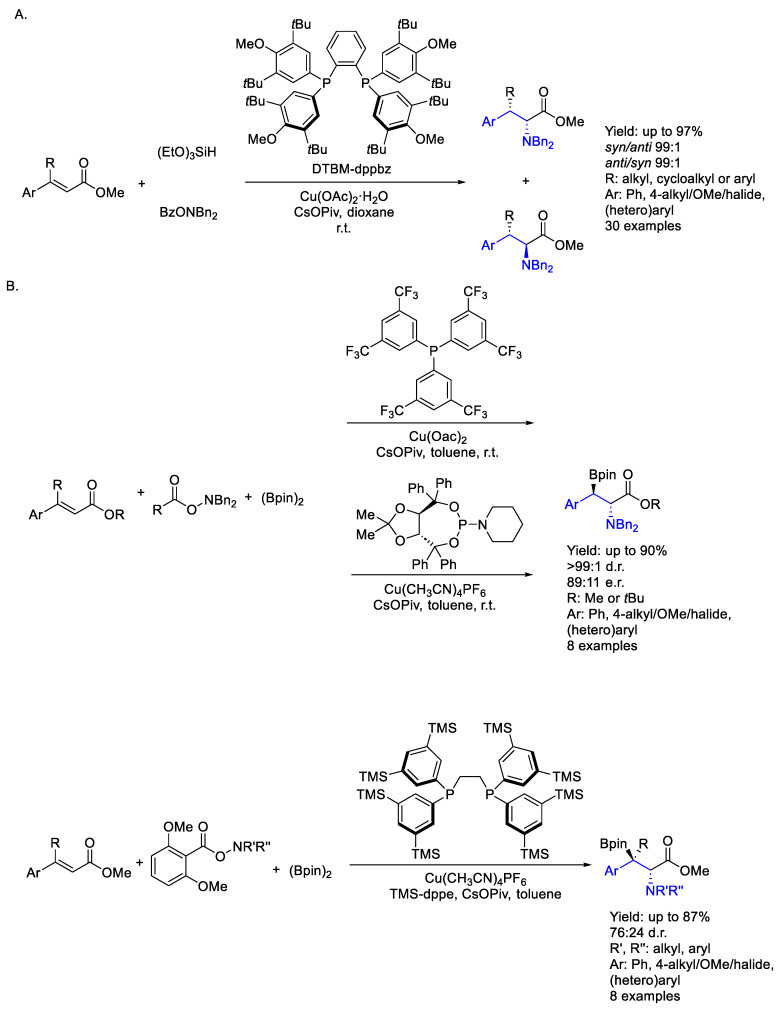

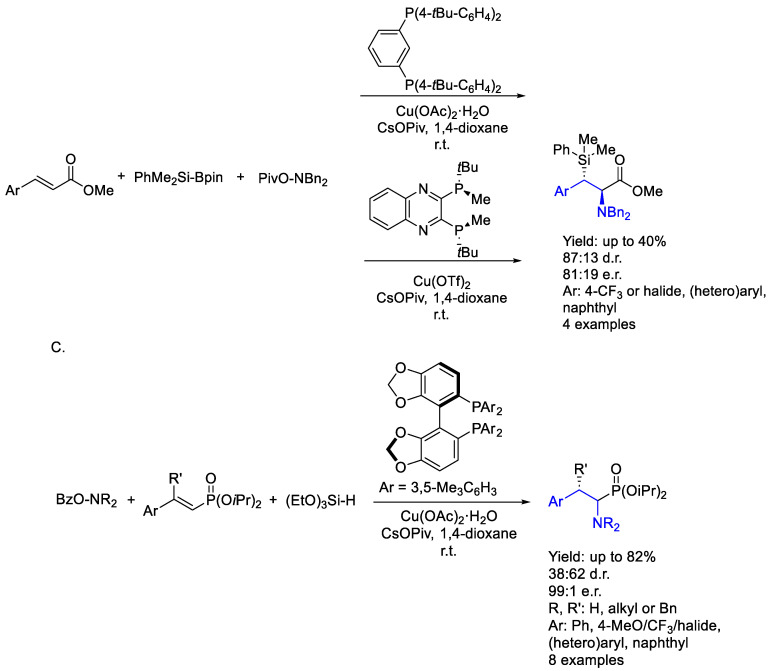
Chiral synthesis of AEA by copper catalysis by Hirano et al. in 2021 (**A**), 2022 (**B**), and 2023 (**C**).

Following this copper-catalyzed asymmetric synthesis of AEA, Hirano et al. have shown great success in recent years. Starting in 2021 [31], they developed the regioselective synthesis of α-aminoacid by asymmetric hydroamination of α,β-unsaturated carbonyls enabled by an organo-copper complex. This resulted in an interesting AEA that incorporated ester groups into its structure (Scheme 14A). The key to regioselectivity control was the use of hydroxylamine as an umpolung, electrophilic amination reagent, and remote steric hindrance by both the chiral catalyst and DTBM-dppbz ligand. One year later [32], they published back-to-back studies based on the synthesis of α-amino acids by either borylamination or silylamination reations enabled by carefully chosen bulky chiral organo-copper complexes, synthesizing a large variety of chiral AEAs and achieving great success in both yields and ee, with α-substitution of either boron or silane derivatives (Scheme 14B). Finally, in 2023 [33], they reported the asymmetric synthesis of α-aminophosphonates by copper-catalyzed regioselective hydroamination of α,β-unsaturated phosphonates. Following their previous strategies, an umpolung, electrophilic amination with the hydroxylamine resulted in an interesting chiral AEA in great ee and moderate dr (Scheme 14C).

**Scheme 15:**
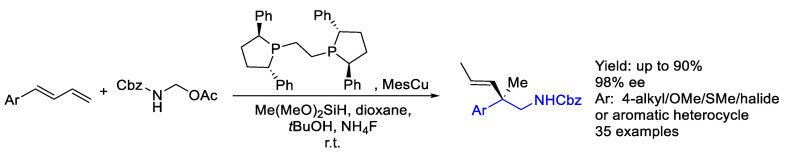
Chiral AEA from 1,3-dienes and *N*,*O*-acetals synthesized in 2023 by Yu et al.

Following that year’s studies, Yu et al. [34]. developed the synthesis of interesting β-chiral amines with quaternary stereocenters by copper-catalyzed reductive aminomethylation of 1,3-dienes with *N*,*O*-acetals, resulting in a chiral AEA with high chemo-, regio-, *E*/*Z*- and enantioselectivities, as shown in Scheme 15.

**Scheme 16:**
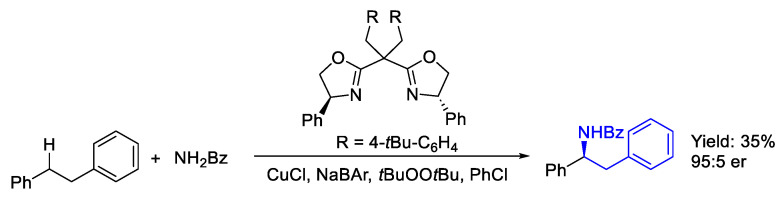
Single AEA synthesized by Zhou et al.’s methodology in 2023.

In 2023, Zhou et al. [35] developed a methodology in which an intermolecular enantioselective benzylic C(sp^3^)-H amination by cationic copper catalysis occurred, synthesizing useful α-phenethylamines (Scheme 16). Mechanistic studies suggested that the amination likely proceeded via a radical mechanism in which C–H bond cleavage was the rate-limiting step. In this process, an interesting chiral β-phenyl phenethylamine was produced in moderate yield and great ee.

**Scheme 17:**
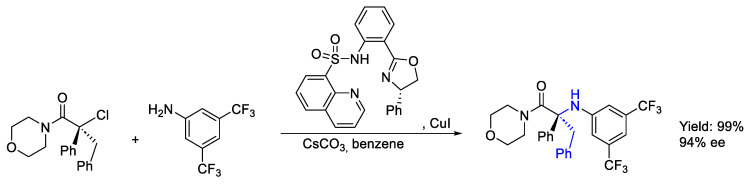
Chiral quaternary carbon containing AEA by Liu et al. in 2023.

Also in 2023, Liu et al. [36]. reported a copper-catalyzed enantioconvergent radical C(sp^3^)–N cross-coupling of activated racemic alkyl halides with (hetero)aromatic amines, resulting in asymmetric quaternary carbon AEA (Scheme 17). Copper catalysis in combination with multidentate anionic ligands led to excellent yields and ee, as this kind of ligand could not only enhance the reducing capability of the copper catalyst to provide an enantioconvergent radical pathway but also avoid coordination with other coordinating heteroatoms, thereby overcoming catalyst poisoning and/or chiral ligand displacement.

**Scheme 18:**
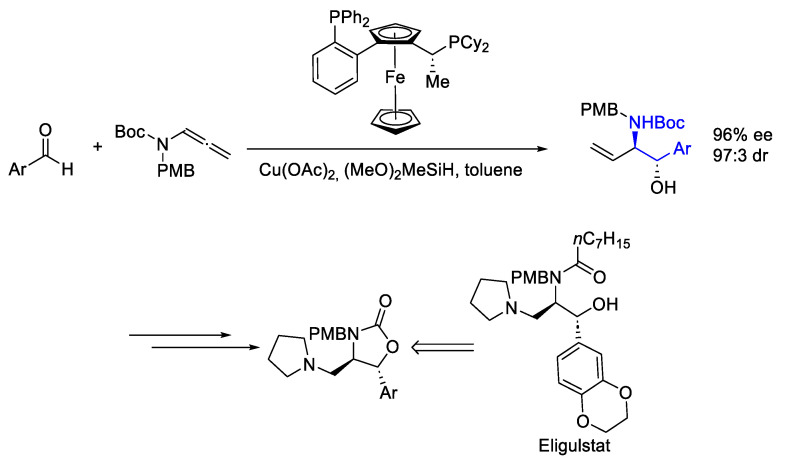
Asymmetric synthesis of AEAs and their derivatization towards an eliglustat precursor by Sieber et al.

Ending that year’s research, Sieber et al. [37] reported an interesting synthesis of chiral 1,2-aminoalcohols through enantioselective copper-catalyzed reductive coupling of aldehydes and allenamides, overcoming the problematic competitive reduction of the aldehyde electrophile by a CuH catalyst. Enantiopure vinyl AEAs were synthesized in great ee and dr. Also, subsequent derivatization of the products could lead to key intermediate in the synthesis of eliglustat, a treatment for Gaucher’s disease (Scheme 18).

**Scheme 19:**
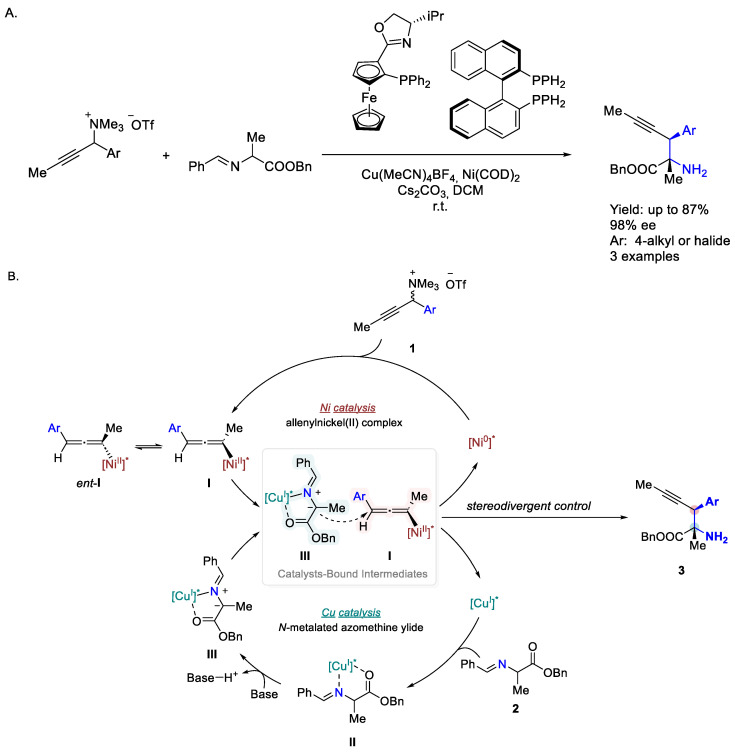
Chiral quaternary carbon containing AEA (**A**) and proposed dual catalysis mechanism (**B**) by Guo et al.

Later, in 2024, Guo et al. [38] reported the synthesis of an interesting chiral AEA by dynamic kinetic stereodivergent transformation of racemic propargylic ammonium salts enabled by dual copper and nickel catalysis. The resulting α-quaternary amino ester containing an optically active AEA was synthesized via C–N bond cleavage in good yields and ee (Scheme 19A). A plausible mechanism was proposed, in which nickel catalysis intertwined with copper catalysis for the DyKAT of racemic propar-gylic ammonium salts with prochiral aldimine esters. Thus, chiral nickel complex cleavaged C–N bonded to generate the electrophilic allenylnickel(II) intermediates. Subsequent nucleophilic addition of the copper-coordinated azomethine ylides onto the allenylnickel(II) intermediates via a catalytic enantio–convergent pathway would provide a novel stereoselective route to access chiral α-tertiary amines through the propargylation process (Scheme 19B).

**Scheme 20:**
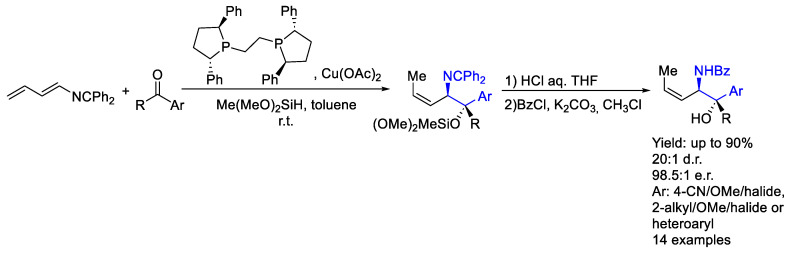
Chiral 1,2-aminoalcoholes by Macolmson et al. in 2024.

Also in 2024, Malcolmson et al. [39] developed a methodology for the (*Z*)-selective aminoallylation of a range of ketones to prepare allylic 1,2-amino tertiary alcohols. Thus, copper-catalyzed reductive couplings of 2-azatrienes with aryl/alkyl and dialkyl ketones proceed with Ph-BPE as the supporting ligand, generating *anti*-amino alcohols with >98% (*Z*)-selectivity under mild conditions. Thus, subsequent derivatization of the corresponding products afforded a range of chiral AEAs in excellent yields and ee (Scheme 20).

**Scheme 21:**
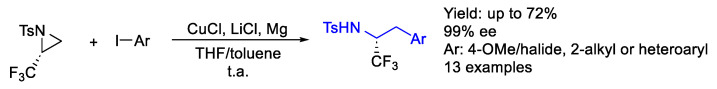
Single chiral AEA through copper catalysis by Lee et al. in 2024.

The same year, Lee at al. [40] reported an interesting regioselective and stereospecific aryl Grignard addition for the copper-catalyzed trifluoromethyl aziridine opening. Desired products could be derivatized in order to obtain interesting azaindoles. An achiral copper complex was used for the ring opening of optically active activated aziridines with aryl iodides in order to synthesize an asymmetric AEA in good yields and maintaining the ee (Scheme 21).

**Scheme 22:**
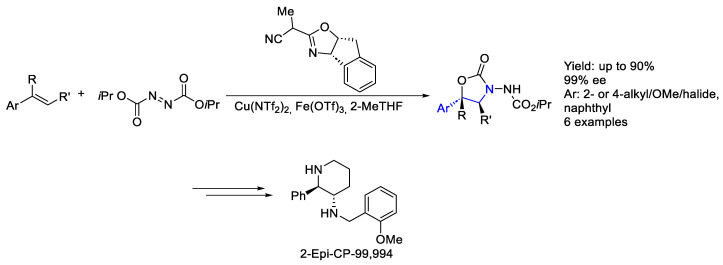
Polisubstituted chiral AEAs and their derivatization by Zhao et al. in 2024.

Finally, Zhao et al. [41] reported the enantioselective aminative difunctionalization of alkenes via copper-catalyzed electrophilic addition with nitrogen sources, synthesizing cyclic hydrazine derivatives via either [3 + 2] cycloaddition or intramolecular cyclization in high chemo-, regio-, enantio-, and diastereoselectivities. By using a chiral organo-copper complex, interesting polisubstituted chiral AEAs were produced in excellent yields and ee. Also, subsequent derivatization could lead to potent orexin receptor antagonist 2-Epi-CP-99,994 (Scheme 22).

### 2.2. Rhodium

**Scheme 23:**
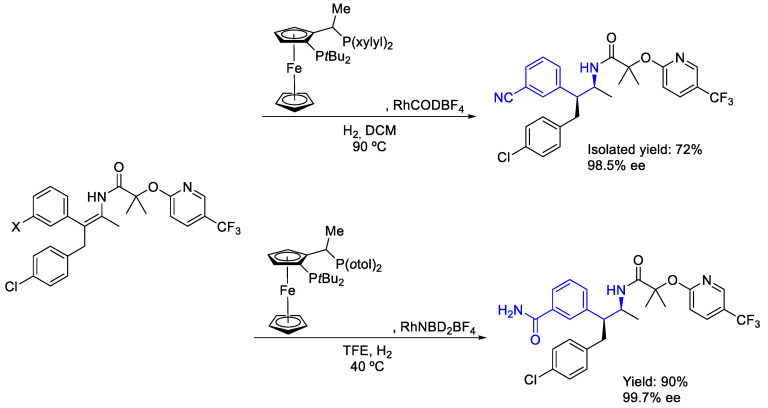
Chiral AEA by rhodium catalysis by Wallace et al. in the synthesis of taranabant.

Starting in 2009, Wallace et al. [42] reported the asymmetric synthesis of the bioactive compound taranabant, a CB1R inverse agonist in the treatment of obesity. The key step in the introduction of chirality was the asymmetric hydrogenation of the corresponding substrate enabled by an organo-rhodium catalyst based on a ferrocene moiety, leading to a couple of chiral AEAs in great yields and ee (Scheme 23).

**Scheme 24:**
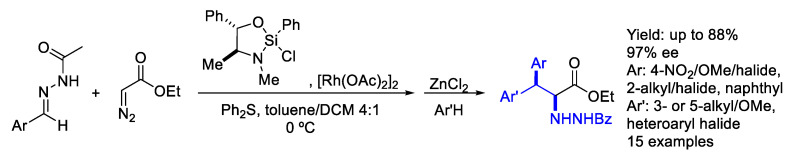
Asymmetric biaryl AEA by Leighton et al. in 2009.

In the same year, Leighton et al. [43] developed the asymmetric synthesis of an interesting chiral AEA in which the nitrogen atom is included in a hydrazine group. This rhodium–zinc dual catalyzed tandem asymmetric aza-arzens/ring-opening reaction led to excellent yields and ee, affording interesting α-amino esters as products that could be derivatized to obtain the corresponding free amine substrates (Scheme 24). Interestingly, the chiral silane Lewis acid performs two distinct functions, activating the initially formed aziridine toward ring-opening reactions with either chloride or arene nucleophiles to deliver complex amino acid derivatives in a simple one-pot process.

**Scheme 25:**
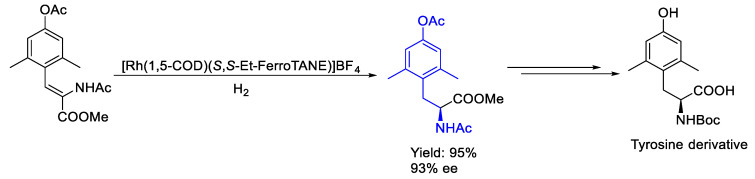
Asymmetric tyrosine derivative through rhodium catalysis by Praquin et al. in 2011.

Two years later, in 2011, Praquin et al. [44] developed an asymmetric hydrogenation route to (*S*)-*N*-Boc-2,6-dimethyltyrosine. The discovery of a new chiral organo-rhodium complex based on a ferrocene moiety was the key step in the improvement of the previously known synthetic routes. Thus, the corresponding substrate was hydrogenated in excellent ee and yield, leading to an interesting tyrosine derivative (Scheme 25).

**Scheme 26:**
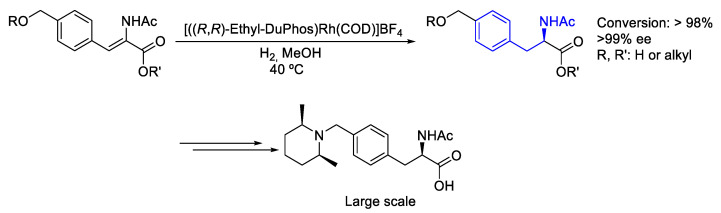
Large-scale synthesis of phenylalanine derivative by Fox et al. in 2011.

In the same year, Fox et al. [45] reported large-scale synthesis of a phenylalanine derivative by asymmetric hydrogenation using a chiral organo-rhodium catalyst from the corresponding enamine substrate. This multi-gram reaction could afford the desired product in excellent yield and ee (Scheme 26)

**Scheme 27:**
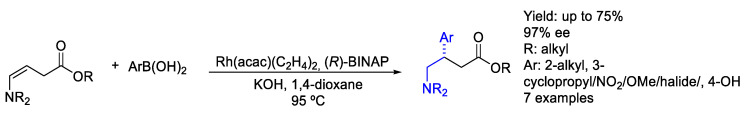
Asymmetric synthesis of γ-aminobutirc acid derivatives by Anderson et al. in 2012.

One year later, Anderson et al. [46] reported the direct addition of arylboronic acids to unsaturated esters containing basic γ-amino groups. A Miyaura–Hayashi-based synthetic route was developed using asymmetric rhodium catalysis, obtaining chiral AEAs as γ-aminobutirc acid derivatives in good yields and ee (Scheme 27).

**Scheme 28:**
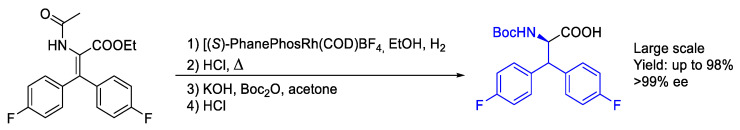
Multi-kilogram scale symmetric synthesis of AEA by Ramsden et al. in 2012.

Also in 2012, Ramsden et al. [47] developed a multi-kilogram reaction towards the synthesis of (*S*)-*N*-Boc-bis(4-fluorophenyl)alanine, by asymmetric hydrogenation of the corresponding substrate using rhodium catalysis (Scheme 28). They achieved the manufacture of 900 kg of the desired product in excellent yield and ee by subsequent isolations.

**Scheme 29:**
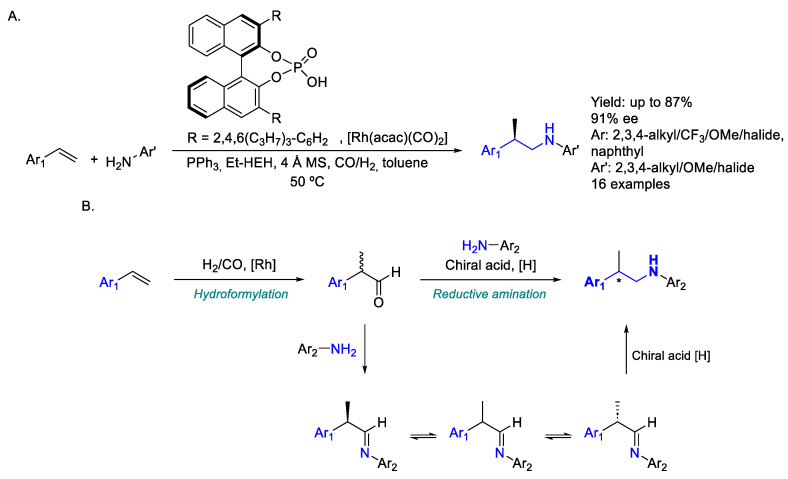
Asymmetric synthesis of AEA (**A**) by DKR process (**B**) by Xiao et al. in 2015.

In 2015, Xiao et al. [48] reported an asymmetric synthesis of an α-substituted chiral AEA. This asymmetric hydroaminomethylation of styrenes was enabled by a rhodium–phosphine species and a chiral phosphoric acid and afforded good yields and ee (Scheme 29A). The transformation was made possible by combining metal- and organocatalysis, with the former catalyzing the hydroformylation while the latter catalyzed reductive amination via DKR, as shown in Scheme 29B.

**Scheme 30:**
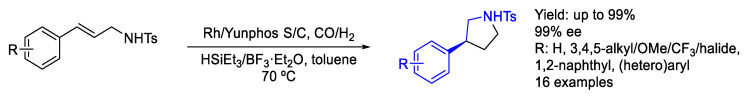
Pyrrolidine-containing chiral AEA by Zhang et al. in 2016.

In 2016, Zhang et al. [49] reported the first interrupted asymmetric hydroaminomethylation reaction, obtaining an interesting chiral AEA in which the nitrogen atom is embedded into a pyrrolidine ring. Starting from *trans*-1,2-disubstituted olefins, the resulting enantiopure products were obtained in excellent yields and ee using a chiral organo-ruthenium complex (Scheme 30). The key step in the synthesis was trapping the resulting hemiaminal intermediate by either oxidation with PCC or reduction with boron species, ending in the desired compounds.

**Scheme 31:**
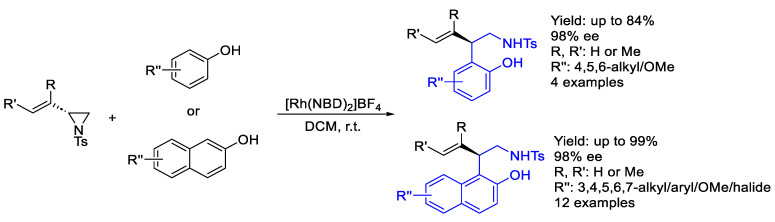
Asymmetric AEA synthesized in 2017 by Feng et al. through rhodium catalysis.

In 2017, Feng et al. [50] reported a rhodium-catalyzed chemoselective and regioselective allylic alkylation of hydroxyarenes with vinyl aziridines. Starting from the corresponding optically active aziridine substrates, this methodology afforded an interesting wide range of chiral 2-vinyl-2-arylethylamine derivatives in good yields and ee by taking advantage of chirality-transfer strategy. Thus, this methodology demonstrated that hydroxyarenes can serve as a *C*-nucleophiles rather than *O*-nucleophiles in rhodium-catalyzed regioselective ring-opening reactions of vinylaziridines (Scheme 31).

**Scheme 32:**
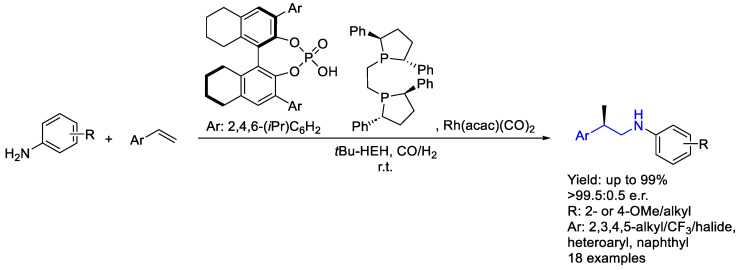
Chiral AEA enabled by rhodium catalysis by Hen et al. in 2017.

Han et al., in 2017 [51], reported the synthesis of chiral α-methyl AEA in excellent yields and ee by using a rhodium catalyst with a commercial ligand and a phosphoric acid catalyst by asymmetric hydroaminomethylation enabled by rhodium-complex formation, which has been proved to be a very good strategy in order to synthesize chiral AEAs (Scheme 32). The relay catalytic reaction consisted of a rhodium-catalyzed hydroformylation step and Brønsted acid-catalyzed subsequent dynamic kinetic reductive amination process.

**Scheme 33:**
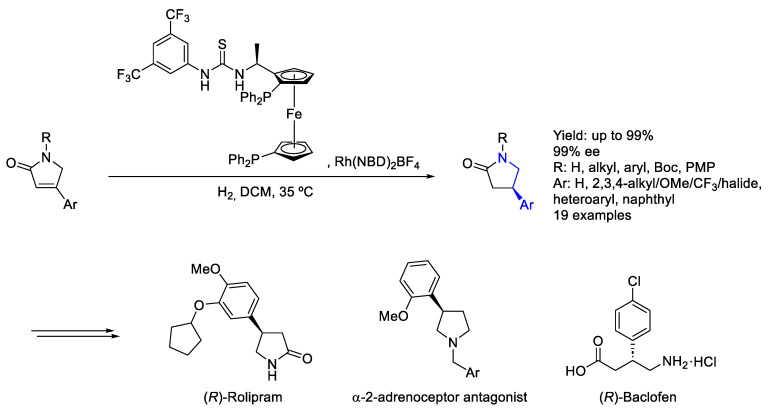
Chiral γ-lactam and their derivatization towards bioactive compounds by Zhang et al. in 2018.

One year later, Zhang et al. [52] reported the asymmetric hydrogenation of γ-lactams, introducing great values of enantiopurity by using an organo-rhodium complex based on a ferrocene moiety. This methodology could afford a chiral AEA in which the nitrogen atom is embedded into a chiral γ-lactam ring (Scheme 33). Also, they proved the methodology’s applicability by the synthesis of drug molecules such as rolipram, baclofen, and α-2-adrenoceptor antagonist.

**Scheme 34:**
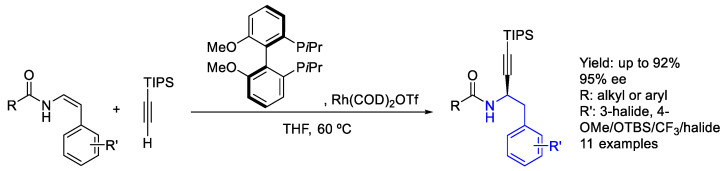
Chiral α-propargyl amide synthesis by Li et al. in 2017.

Also in 2018, Li et al. [53] developed a highly enantioselective synthesis of propargyl amides enabled by rhodium-catalyzed asymmetric hydroalkynylation of enamides. This process afforded a great family of chiral AEAs in excellent yields and ee (Scheme 34). Starting from the corresponding silyl alkyne substrate, this methodology relied on stereospecific hydroalkynylation of the electron-rich alkene, a completely different mechanism with conventional alkynylations that occurred under proton-transfer conditions.

**Scheme 35:**
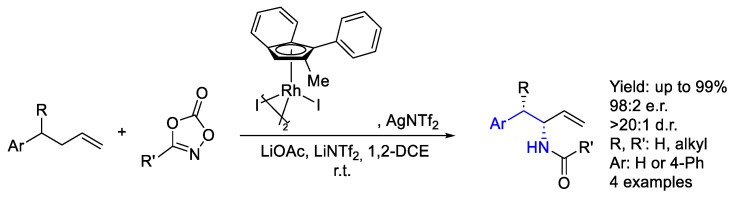
Asymmetric synthesis of β-vinyl AEA by Blakey et al. in 2020.

In 2020, Blakey et al. [54] developed the synthesis of a new organo-rhodium-based catalyst for regio- and enantioselective allylic C–H amidation. Thus, a planar chiral rhodium indenyl complex was successfully used in order to synthesize a chiral AEA by the asymmetric allylic C–H amidation of unactivated olefins, delivering a wide range of high-value enantioenriched AEAs in excellent yields and excellent ee (Scheme 35). Computational studies suggested that C–H cleavage is rate- and enantio-determining, while reductive C–N coupling from the Rh(V)-nitrenoid intermediate is regio-determining.

**Scheme 36:**
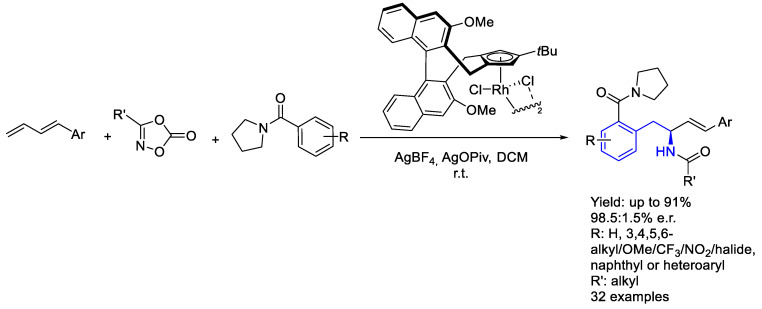
Rhodium-catalysed chiral AEA by Wang et al. in 2021.

In 2021, Wang et al. reported [55] a rhodium-catalyzed enantioselective C–H activation three-component coupling of arene, diene, and dioxazolone towards the synthesis of a chiral allylic AEA, in 1,2-*ortho*-selectivity, *E*-selectivity, and enantioselectivity. This reaction proceeded via a C–H activation pathway en route to thermodynamically stable as well as kinetically reactive π-ally intermediates. Thus, excellent yields and ee were achieved (Scheme 36).

**Scheme 37:**
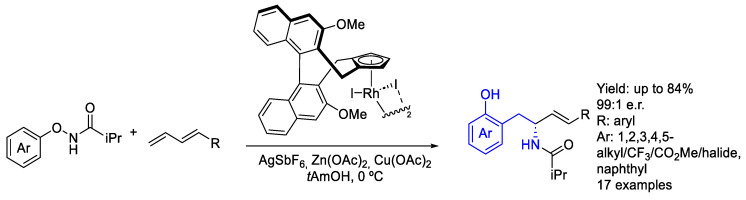
CpXRh(III)-catalyzed asymmetric AEA by Yi et al. in 2021.

The same chiral allylic amine structural motif was also synthesized at the same time and enabled by the same type of organo-rhodium catalyst by Yi et al. [56]. Thus, starting from the corresponding *N*-phenoxy amides, intermolecular aryl C–H activation and intramolecular amide transfer were achieved by CpXRh(III)-catalyzed enantioselective intermolecular carboamination (Scheme 37). Sequential formation of a completely regioselective C–C bond and a highly enantioselective C–N bond occurred in a catalytic pathway in which an alkene insertion/π-allylation/intramolecular nucleophilic substitution cascade was involved.

**Scheme 38:**
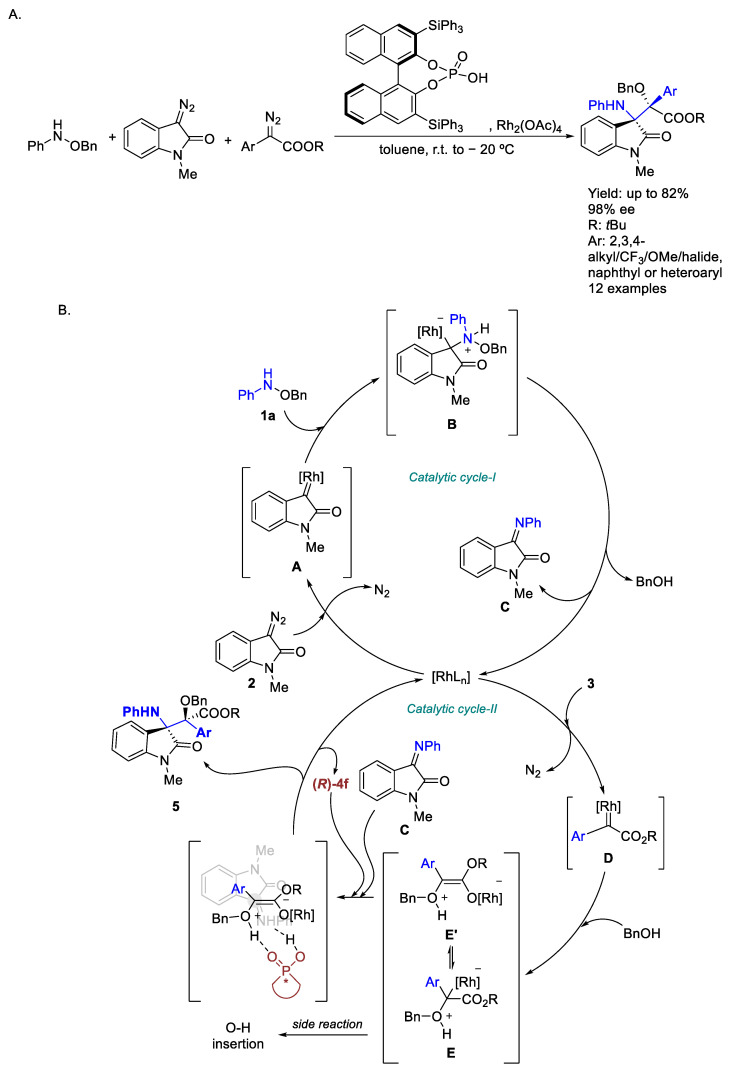
(**A**) Asymmetric synthesis of AEA by rhodium catalysis. (**B**) Proposed dual mechanism. Xu et al., 2020.

One year later, Xu et al. [57] developed an asymmetric three-component reaction towards the synthesis of chiral α-alkoxy-β-amino-carboxylates by rhodium-catalysis, resulting in a polisubstituted asymmetric AEA (Scheme 38A). Thus, asymmetric formal aminohydroxylation of diazo compounds with *O*-benzyl hydroxylamines involving N–O bond activation, fragment modification, and a reassembly cascade process was achieved. This cascade reaction forms multiple bonds in one pot, including C=N, C–O, and C–C bonds, providing a potent complement for the aminohydroxylation, as shown in the proposed double catalytic cycle (Scheme 38B), thus resulting in good yields and ee.

**Scheme 39:**
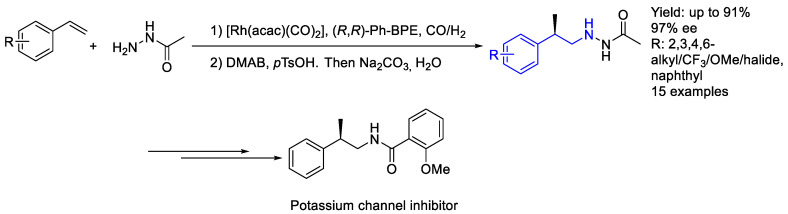
Chiral AEA precursor of potassium channel inhibitor by Franciò et al. in 2022.

Also in 2022, Franciò et al. [58] kept pushing the synthetic methodology of styrene derivatives by hydroaminomethylation reactions enabled by rhodium catalysis when starting from the corresponding styrenes and hydrazides. This methodology could afford a chiral AEA in which the nitrogen atom forms either hydrazones, hydrazides, hydrazines, or amines, in excellent yields and ee. Subsequent derivatization of the products could lead to the synthesis of interesting bioactive compounds, such as a potassium channel inhibitor (Scheme 39).

**Scheme 40:**
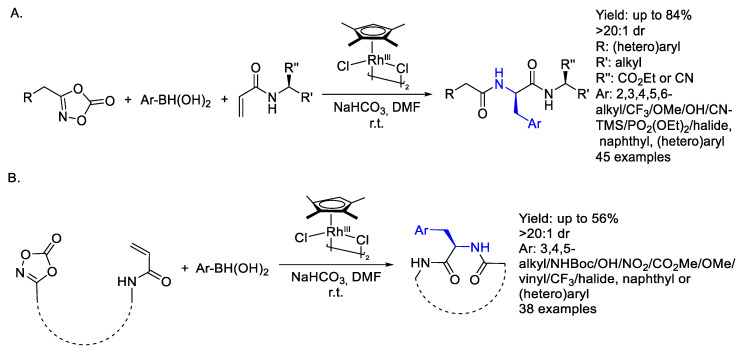
Chiral AEA containing polypeptide synthesis through rhodium catalysis by Rovis et al. in 2022 (**A**) and 2024 (**B**).

Novel developments in the synthesis of unnatural peptides came from the studies of Rovis et al. In 2022 [59], they achieved the modular synthesis of peptides by a diastereoselective three-component carboamidation reaction enabled by an organo-rhodium complex. Starting from the corresponding dioxazolones, arylboronic acids, and acrylamide derivatives, new amide bonds were formed towards the synthesis of an interesting chiral AEA embedded into a peptide chain in good yields and ee (Scheme 40A). Two years later, in 2024 [60], they achieved the same level of success by using this methodology to obtain unnatural peptide macrocycles, starting from four to fifteen amino acid chains, incorporating chiral AEA motives towards three-component macrocyclation, achieving 15-mer macrocyclic substrate reaction (Scheme 40B).

**Scheme 41:**
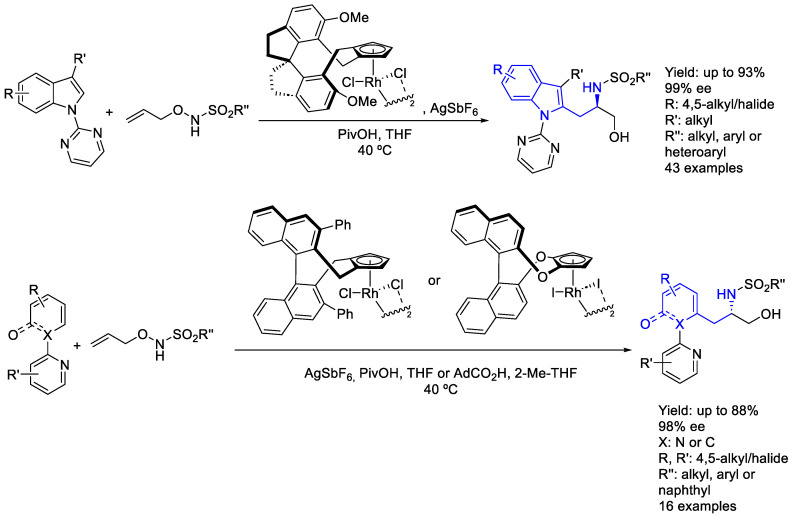
Rhodium catalysts used by the asymmetric synthesis of AEA by Li et al. in 2023.

In 2023, Li et al. [61] reported the asymmetric synthesis of an AEA which included axially and centrally chiral amino alcohols starting from interesting *O*-allylhydroxyamine derivatives. The key step was the C=C bond in *O*-allylhydroxyamine activation by the intramolecular electrophilic amidating moiety, as well as a migrating directing group. This methodology could afford a polisubstituted chiral AEA controlling the stereochemistry of the process by switching between chiral organo-rhodium complex; and, by the employment of axially prochiral or axially racemic heteroarenes, it afforded amino alcohols with both axial and central chirality in excellent enantio- and diastereoselectivity (Scheme 41).

**Scheme 42:**
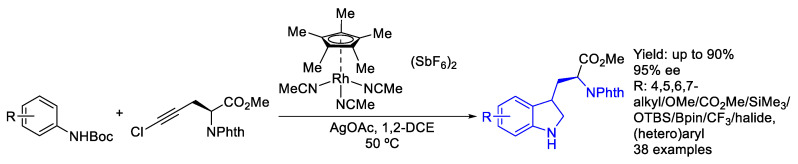
Non-Canonical tryptophan synthesis by Seyedsayamdost et al. in 2024.

In 2024, Seyedsayamdost et al. [62] developed the synthesis of non-canonical tryptophan derivatives by C-H functionalization of anilines enabled by organo-rhodium catalysis. This regioselective synthesis afforded C4-C7 substituted tryptophan derivatives enabled by catalyzed annulation between structurally diverse *tert*butyloxycarbonyl(Boc)-protected anilines and alkynyl chlorides, achieving great success in yields and ee (Scheme 42).

**Scheme 43:**
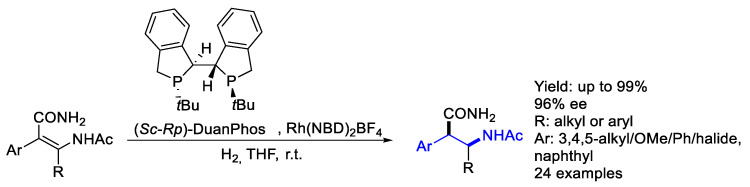
Asymmetric synthesis of AEA through rhodium catalysis by Lv et al. in 2024.

Also in 2024, Lv et al. [63] developed the synthesis of chiral β-aminoamides by asymmetric hydrogenation of the corresponding tetrasubstituted α,β-unsaturated amide. Using an organo-rhodium complex, the resulting chiral AEAs with two contiguous chiral centers were synthesized in excellent yields and ee. Also, gram-scale reaction and efficient transformation of β-amino amide to β-amino acid and β-amino cyanide were achieved (Scheme 43).

**Scheme 44:**
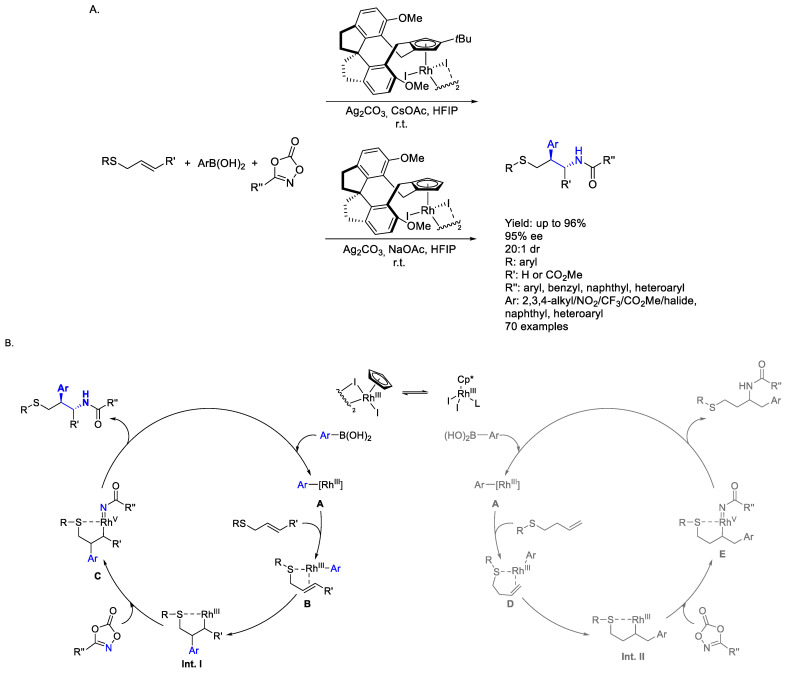
Amide-containing chiral AEA synthetized by Wang et al. in 2024 (**A**) and its proposed mechanism (**B**).

Finally, Wang et al. [64] reported a diastereo- and enantioselective regiodivergent (hetero)arylamidation of (homo)allylic sulfides enabled by Rh(III) catalysis. This three-component reaction methodology could afford chiral AEAs in excellent yields, ee and dr starting from the corresponding homoallyl sulfide, aryilboronic acid, and dioxazolone derivatives (Scheme 44A). Although an asymmetric AEA could be synthesized by following one of the two possible catalytic pathways, a sulfide moiety plays an important role in the reaction mechanism, promoting migratory insertion and controlling the regioselectivity of the reaction, as shown in Scheme 44B.

### 2.3. Palladium

**Scheme 45:**
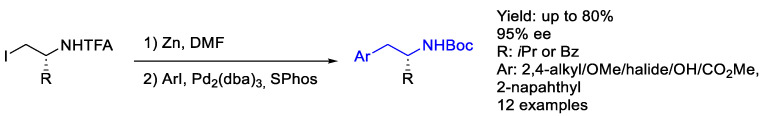
Chiral AEA by Jackson et al. in 2010 through palladium catalysis.

Starting in 2010, Jackson et al. [65] developed the synthesis of interesting chiral AEA derivatives by a Negishi cross-coupling reaction starting from enantiopure alkyl iodide and aryl iodide substrates using an organo-palladium complex. The resulting chiral AEAs were synthesized, maintaining the stereochemistry in good yields (Scheme 45).

**Scheme 46:**
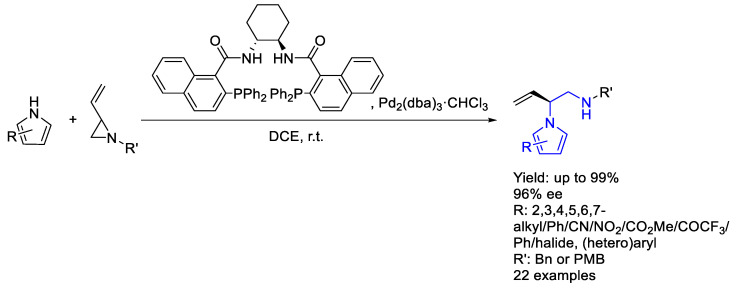
Interesting asymmetric AEA by Trost et al. in 2010.

In the same year, Trost et al. [66] reported a palladium-catalyzed dynamic kinetic asymmetric alkylation of vinyl aziridines. Starting from both substituted 1*H*-pyrroles and 1*H*-indoles, this high regio-, chemo-, and enantioselective process could afford an interesting chiral AEA in excellent yields and ee. Interestingly, the obtained products could be derivatized to obtain bioactive compounds (Scheme 46).

**Scheme 47:**
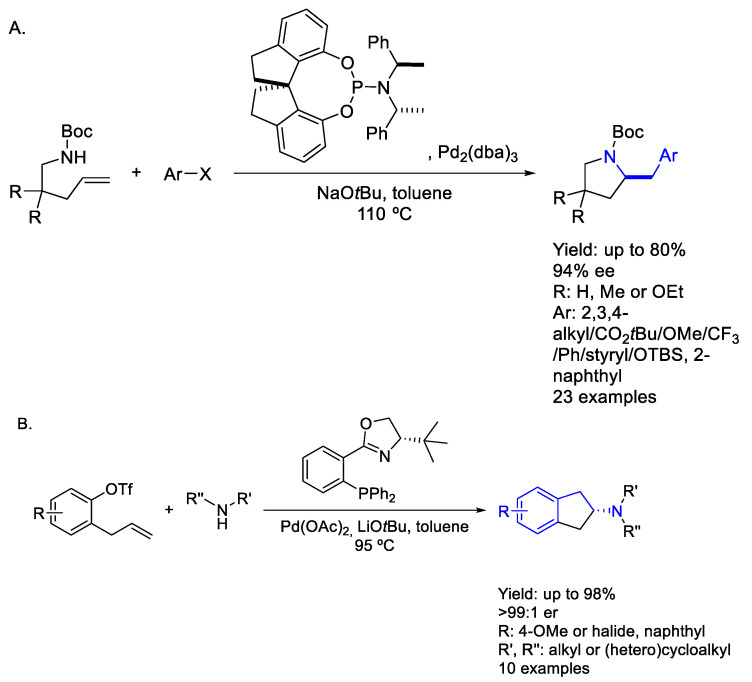
Palladium-catalysed chiral AEA by Wolfe et al. in 2010 (**A**) and 2015 (**B**).

Also in 2010, Wolfe et al. [67] achieved the synthesis of an enantioenrich AEA in which the nitrogen atom is embedded into a tetrahydropyrrole ring. Starting from readily available alkenyl or aryl bromides and *N*-boc-pent-4-enylamines and using a chiral organo-palladium complex, they reported high yields and ee (Scheme 47A). Five years later [68], they developed the asymmetric synthesis of 2-aminoindane derivatives by asymmetric palladium-catalyzed alkene carboamination reactions. In this way, interesting chiral AEAs were synthesized in excellent yields and ee (Scheme 47B).

**Scheme 48:**
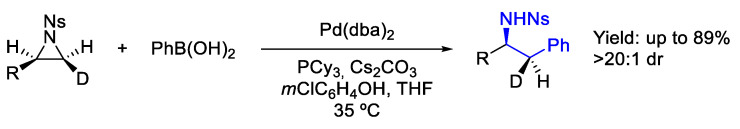
Cross-coupling reaction catalyzed by a palladium complex towards the synthesis of a chiral AEA by Michael et al. in 2013.

In 2013, Michael et al. [69] developed an interesting methodology to synthesize substituted chiral phenethylamines by a cross-coupling reaction of unsubstituted and 2-alkyl-substituted aziridines with arylboronic acid nucleophiles using a palladium complex. Starting from enantiopure activated aziridines, this reaction afforded an AEA that maintained the stereochemistry in good yields (Scheme 48).

**Scheme 49:**
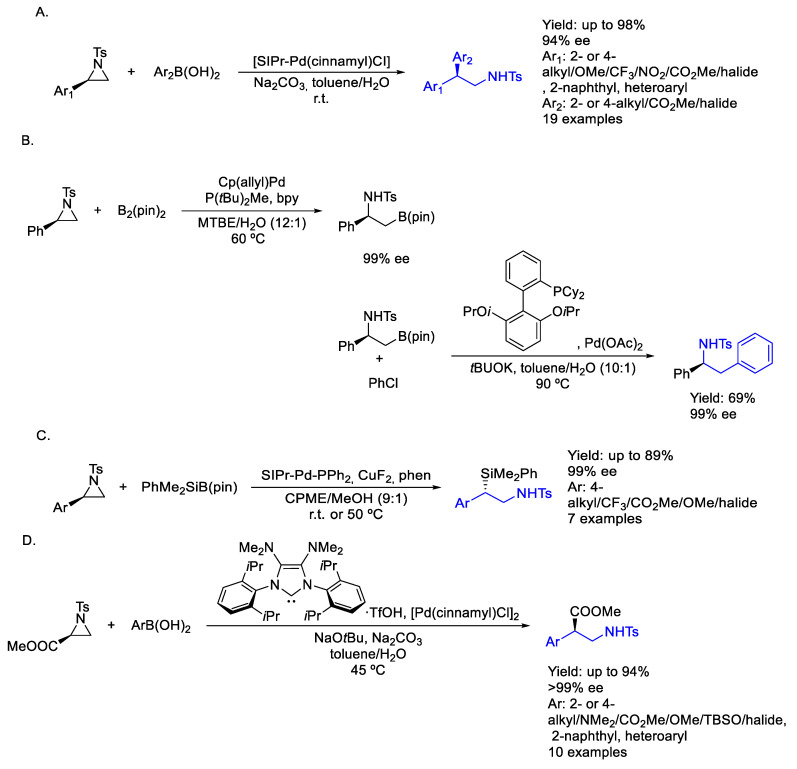
Chiral palladium catalysis methodology by Minakata et al. towards the synthesis of biarylethylamines in 2014 (**A**), phenethylamines in 2016 (**B**) and silyl-containing AEA in 2019 (**C**) and AEA based on beta-amino esters (**D**) in 2019.

When it comes to the asymmetric synthesis of AEAs enabled by organo-palladium catalysis, Minakata et al. have shown great success in this field. Starting in 2014 [70], they developed a methodology towards the synthesis of chiral 2-arylphenethylamine derivatives by Pd/NHC-catalyzed enantiospecific and regioselective Suzuki–Miyaura arylation of 2-arylaziridines (Scheme 49A). Starting from enantiopure substrates, they achieved great numbers when it came to yields, maintaining the enantiopurity. Then, in 2016 [71], they increased the substrate scope and developed a methodology starting from enantioenrich aziridine and bis(pinacolato)diboron towards palladium-catalyzed regioselective and stereo-inversive ring-opening borylation of 2-arylaziridines, resulting in interesting products that could be derivatized in order to obtain a chiral AEA in good yield and ee (Scheme 49B). Later, in 2019 [72], by synergistic palladium/copper dual catalysis, starting from chiral activated aziridines and silylborane, they achieved a regiodivergent and stereospecific ring-opening C(sp^3^)–Si cross-coupling reaction leading to an interesting silicon-containing chiral AEA in good yields and ee (Scheme 49C). Also in 2019 [73], they developed a palladium-catalyzed enantiospecific and regioselective ring-opening Suzuki–Miyaura arylation of aziridine-2-carboxylates by a cross-coupling reaction. Starting from enantioenriched substrates, a ring-opening reaction with arylboronic acids led to interesting chiral AEAs in excellent yields and ee (Scheme 49D).

**Scheme 50:**
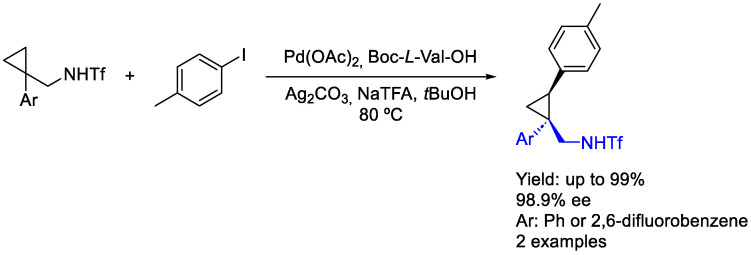
Cyclopropane-based chiral AEA by Yu et al. in 2015.

In 2015, Yu et al. [74] introduced chirality in an already-formed racemic AEA by palladium(II)-catalyzed highly enantioselective C–H arylation of cyclopropylmethylamines. Using aryl iodides as substrates and mono-*N*-protected amino acid ligands, they synthesized several chiral AEAs in excellent yields and ee (Scheme 50).

**Scheme 51:**
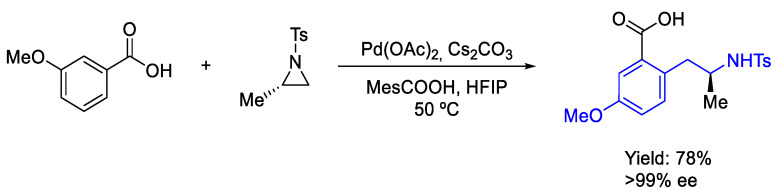
Asymmetric AEA by Zhao et al. in 2019 through palladium catalysis.

Then, in 2019, Zhao et al. [75] used aliphatic aziridines to enable late-stage functionalization of aromatic acids, leading to an interesting AEA by using an organo-palladium-complex via C–H activation (Scheme 51). When it comes to asymmetric synthesis, starting from enantiopure methyl substituted aziridine, the synthesis of a chiral AEA was achieved in good yield and ee.

**Scheme 52:**
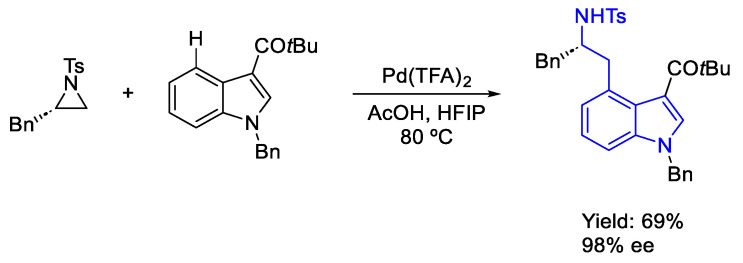
Single chiral AEA synthesized by Koley et al. in 2022.

Later, in 2022, Koley et al. [76] developed a methodology towards the synthesis of β-indolylethylamines by using a palladium complex as a catalyst by a cross-coupling reaction of aliphatic aziridines. Starting from the corresponding indole and enantioenriched aziridine, they could synthesize a single AEA in which the aryl group was an indole, in good ee and yield (Scheme 52).

**Scheme 53:**
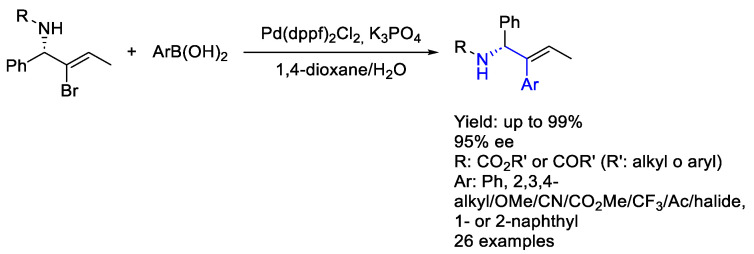
Asymmetric AEA achieved through palladium catalysis by Stecko et al. in 2023.

In 2023, Stecko et al. reported a two-step approach to α-amino ketones involving cross-coupling and an oxidative cleavage sequence, synthesizing a wide variety of chiral AEAs in the process, as key intermediates in the synthesis of α-aminoketones (Scheme 53). Using a palladium complex as a catalyst and starting from chiral amines, they achieved great numbers when it came to yields and ee.

**Scheme 54:**
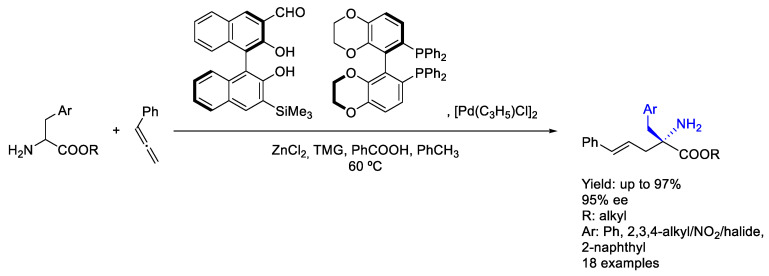
Quaternary-center AEA by Guo et al. in 2023.

In the same year, Guo et al. [77] developed a methodology using modular chiral-aldehyde/palladium catalysis towards the synthesis of an interesting chiral AEA. Starting from racemic α-amino esters and phenylallene, they achieved excellent yields and ee (Scheme 54).

**Scheme 55:**
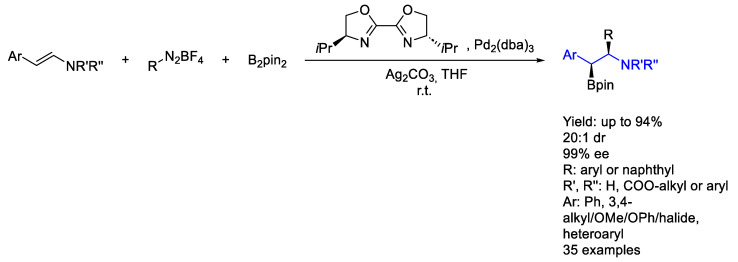
Chiral AEA by Chen et al. in 2024 obtained through palladium catalysis.

Finally, also in 2024, Chen et al. [78] reported a Pd(0)-catalyzed diastereoselective and enantioselective intermolecular Heck–Miyaura borylation of internal enamides. By using a chiral organo-palladium complex, three-component reactions were achieved, resulting in a polisubstituted boron-containing chiral AEA in excellent yields and ee (Scheme 55).

### 2.4. Ruthenium

**Scheme 56:**
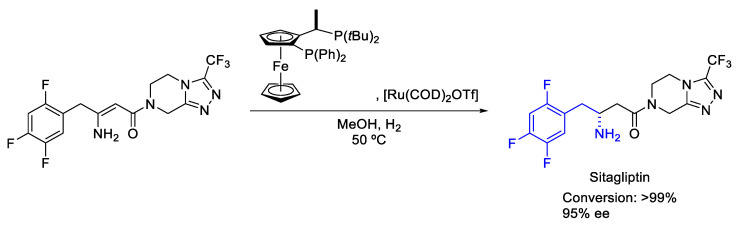
Sitagliptin synthesis by Xu et al. in 2009.

In 2009, Xu et al. [79] developed a promising methodology to synthesize an interesting chiral AEA, sitagliptin, a bioactive molecule used in the treatment of type 2 diabetes, reducing the total waste generated per kilogram and eliminating aqueous waste streams. The process involved asymmetric enamine hydrogenation enabled by an organo-ruthenium complex based on a ferrocene moiety, yielding the desired product in excellent yield and ee (Scheme 56).

**Scheme 57:**
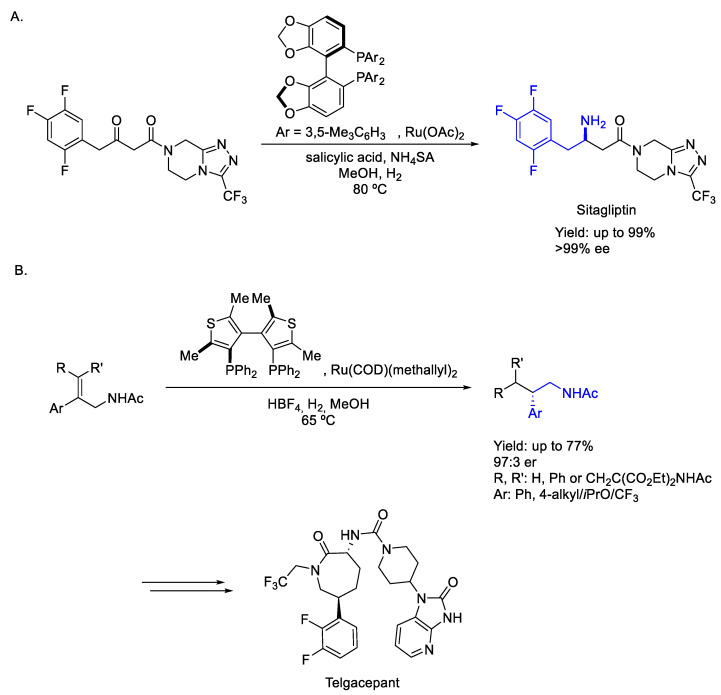
Sitgliptin (**A**) and telgacepant precursor (**B**) by Steinhuebel et al. in 2009 and 2010, respectively.

In this sense, Steinhuebel et al. also obtained sitagliptin by reductive amination of a β-keto amide derivative in a new, high-yield, highly enantioselective synthetic route using a chiral organo-ruthenium complex, also capable of being used in different β-keto amide compounds (Scheme 57A) [80]. Following this study, one year later [81], they also reported the asymmetric hydrogenation of protected allylic amines by ruthenium catalysis, leading to interesting chiral AEAs in excellent yields and ee. Also, a remarkable application of the methodology was shown, as the medicinally relevant bioactive compound telcagepant could be synthesized through this route (Scheme 57B).

**Scheme 58:**
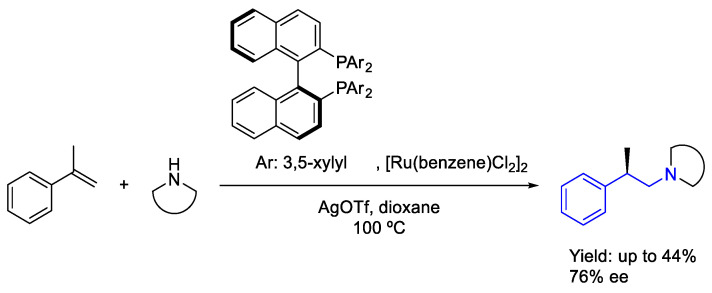
Single chiral AEA by Shibata et al. in 2012.

Later, in 2012, Shibata et al. [82] achieved the asymmetric addition of amines to substituted styrenes by cationic ruthenium complexes combined with diphosphine ligands, with this addition being both β- and enantioselective. Arene-exchange from benzene to α-methylstyrene and subsequent nucleophilic attack towards a previously formed complex were the key steps in the regio- and enantioselection of the reaction. The resulting chiral phenethylamine was afforded in moderate yields and good ee (Scheme 58).

**Scheme 59:**
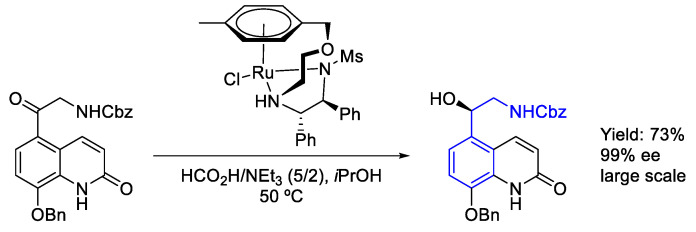
Large-scale synthesis of chiral AEA by Komiyama et al. in 2014.

Two years later, in 2014, Komiyama et al. [83] reported the asymmetric reduction of a key intermediate in the synthesis of β2-adrenergic receptor agonist, involving the reduction of a ketone group by a scalable ruthenium-catalyzed asymmetric transfer hydrogenation, enabled by both the arene/aryl interaction and potential repulsive interaction of the complex between catalyst and substrate (Scheme 59). This process could afford the desired chiral AEA in good yield and ee on a multi-kilogram scale (up to 4.2 kg).

**Scheme 60:**
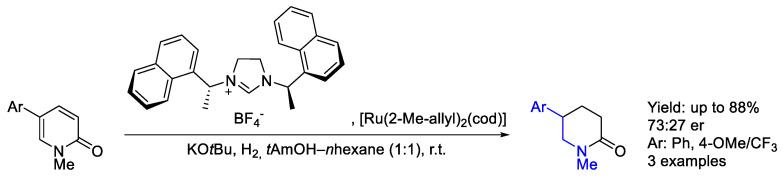
Piperidone-based chiral AEA by Wysocki et al. in 2015.

Later, in 2015, Wysocki et al. [84] developed an asymmetric homogeneous hydrogenation of 2-pyridones using a ruthenium complex with a NHC ligand. The resulting chiral AEAs were synthesized in good yields and moderate ee, leading to compounds in which the nitrogen group was embedded into a piperidone ring (Scheme 60).

**Scheme 61:**
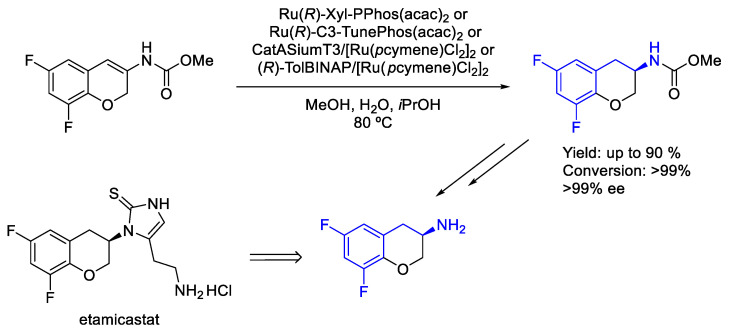
Chiral etimicastat precursor obtained by ruthenium catalysis by Beliaev et al. in 2016.

One year later, Beliaev et al. [85] achieved multi-kilogram-scale asymmetric synthesis of a key intermediate in the production of etamicastat, a chiral AEA peripherally selective dopamine β-hydroxylase inhibitor. The process involved asymmetric hydrogenation of an enamide moiety enabled by several ruthenium-based complexes, affording the desired product in excellent yields and ee (Scheme 61). Their studies allowed identification of four ruthenium-based catalytic systems for asymmetric hydrogenation: two isolated catalysts, Ru(*R*)-Xyl-PPhos(acac)_2_ and Ru(*R*)-C3-TunePhos(acac)_2_, and two preformed non-isolated catalytic systems, CatASiumT_3_/[Ru(pcymene)Cl_2_]_2_ and (*R*)-TolBINAP/[Ru(p-cymene)Cl_2_]_2_. They concluded that the most evident advantage of the isolated ones was lower catalyst loading, as preformation of the catalyst in solution in most cases produced less reactive species, although isolated catalysts were less stable and more oxygen- and moisture-sensitive than the corresponding ligand and metal precursor. On the other hand, considered in terms of commercial efficacy, using low loading of an isolated catalyst may be as convenient as larger amount of a non-isolated one, they concluded.

**Scheme 62:**
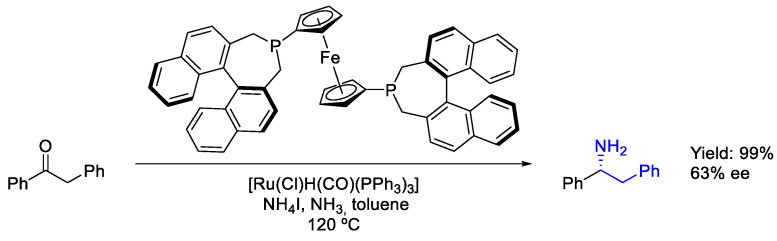
Single chiral AEA obtained by Schaub et al. in 2017.

In 2018, Schaub et al. [86] reported the direct asymmetric reductive amination of alkyl-aryl ketones enabled by ruthenium catalysis with ammonia and hydrogen. The corresponding amines were synthesized in excellent yields and ee. In this process, an interesting phenethylamine was produced (Scheme 62).

**Scheme 63:**
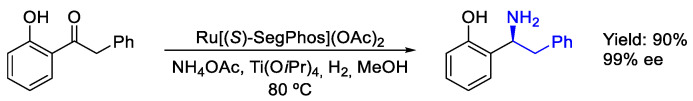
Single chiral diaryl AEA obtained by Yin et al. in 2020.

Two years later, in 2020 [87], Yin et al. published a study in which they performed an asymmetric reductive amination of diaryl and sterically hindered ketones using ammonium salts and hydrogen, enabled by a chiral ruthenium complex. Thus, synthetically useful chiral primary diarylmethylamines and sterically hindered benzylamines were synthesized in excellent yields and ee. In this process, an interesting chiral AEA was produced (Scheme 63).

**Scheme 64:**
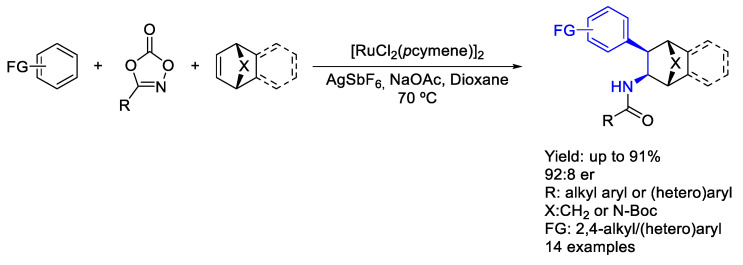
Chiral bicyclic-containing AEA synthesized by Ellman et al. in 2021.

In 2021, Ellman et al. [88] developed a three-component 1,2-carboamidation of bicyclic alkenes, synthesizing an interesting complex chiral AEA. By using a chiral Ru(III) complex, they achieved excellent yields and ee starting from aryl, dioxazolone, and bicyclic substrates. The AEA synthesized in this study consisted of polycyclic compounds in which the nitrogen atom formed an amide group (Scheme 64).

**Scheme 65:**
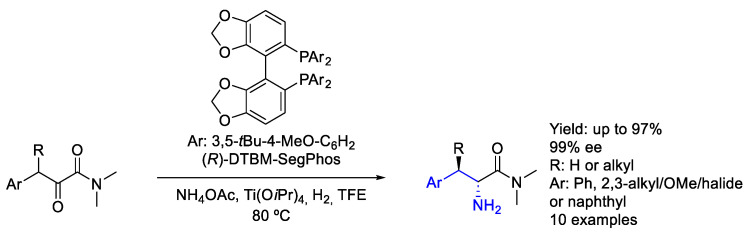
Interesting chiral AEA synthesized in 2022 by Zhang et al.

Later, in 2022, Zhang et al. [89] reported the synthesis of a highly substituted chiral AEA through an unprecedented highly enantioselective ruthenium-catalyzed direct asymmetric reductive amination of α-keto amides with ammonium salts, achieving excellent yields and ee. Using ammonium salts as the nitrogen source, this methodology allowed the synthesis of medicinally interesting *N*-unprotected β-branched α-amino acids containing two contiguous stereogenic centers. Also, they proved the utility of this method through its application in the efficient synthesis of chiral drug intermediates, peptides, and organocatalysts/ligands (Scheme 65).

**Scheme 66:**
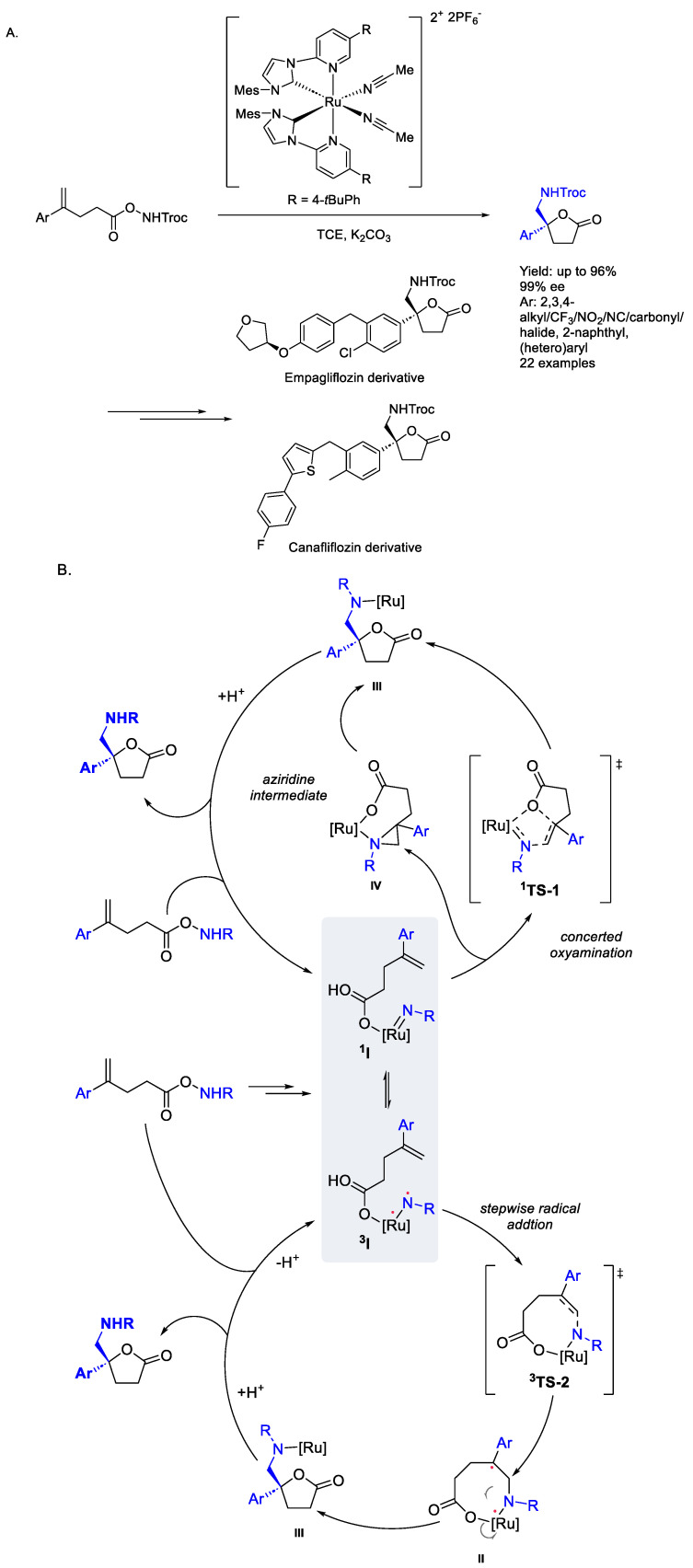
Ruthenium-catalyzed chiral AEA as building block by Meggers et al. (**A**) and its proposed mechanism (**B**).

Later, in 2023, Meggers et al. [90] achieved the synthesis of chiral γ-aminomethyl-γ-lactones, producing an interesting chiral AEA in the process. This methodology involved a nitrene-mediated enantioselective intramolecular olefin oxyamination enabled by a chiral ruthenium complex. Achieving great success in yields and ee, they were also able to derivatize the products, producing interesting building blocks to synthesize bioactive compounds (Scheme 66A). DFT calculations supported the possibility of both a singlet (concerted oxyamination of the alkene) and a triplet pathway (stepwise oxyamination) for the formation of the predominant stereoisomer, as shown in the proposed catalytic cycle. Amine–imine equilibrium led to the two possibilities, resulting in the same enantiomer by either a concerted or a stepwise pathway (Scheme 66B).

**Scheme 67:**
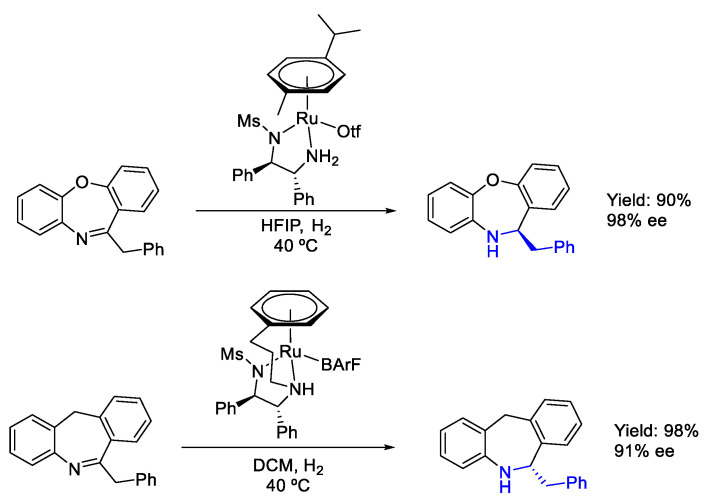
Benzoazepine-based AEA obtained by Chen et al. in 2023.

In the same year, Chen et al. [91] developed an asymmetric hydrogenation of dibenzo-fused azepines using a cationic ruthenium–diamine complex. In this process, an interesting chiral AEA was synthesized, in which the amine group was embedded into an azipine ring, achieving excellent yields and ee (Scheme 67). Interestingly, the enantioselectivities could be regulated by the counteranion of the catalyst in the asymmetric hydrogenation of dibenzo [b,f][1,4]thiazepines and dibenzo[b,e]azepines.

**Scheme 68:**
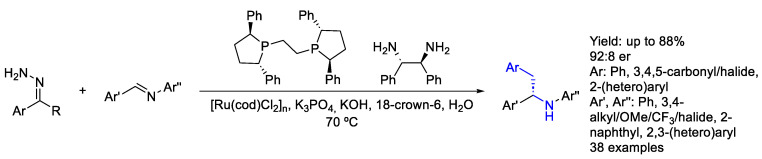
Poliaryl-substituted chiral AEA by Liu et al. in 2023.

Also in 2023, Liu et al. [92] achieved the asymmetric addition of hydrazones to aryl imines in water, by using a ruthenium–diamine–diphosphine system in the presence of crown ether. The methodology used a Ru(II)-catalyzed “umpolung” asymmetric addition of non-substituted hydrazones as an alkyl carbanion equivalent. Thus, poliaryl-substituted chiral AEAs were synthesized in good yields and ee (Scheme 68).

**Scheme 69:**
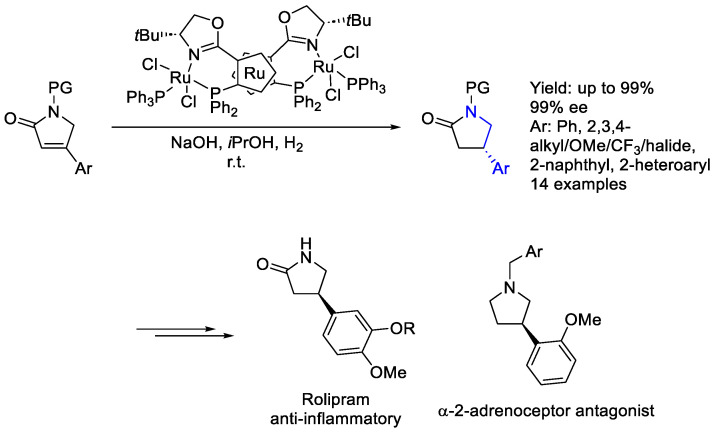
Chiral pyrrolidinone-based AEAs as bioactive compound precursors by Zhang et al. in 2024.

Finally, in 2024, Zhang et al. [93] used a chiral ruthenium complex for the asymmetric hydrogenation of α,β-unsaturated γ-lactams. Thus, when aryl-substituted γ-lactam was used as the starting material, interesting chiral AEAs were synthesized in excellent yields and ee. Also, subsequent derivatization of the products could be undertaken in order to synthesize interesting bioactive molecules, such as rolipram and an α-2-adrenoceptor antagonist (Scheme 69).

### 2.5. Nickel

**Scheme 70:**
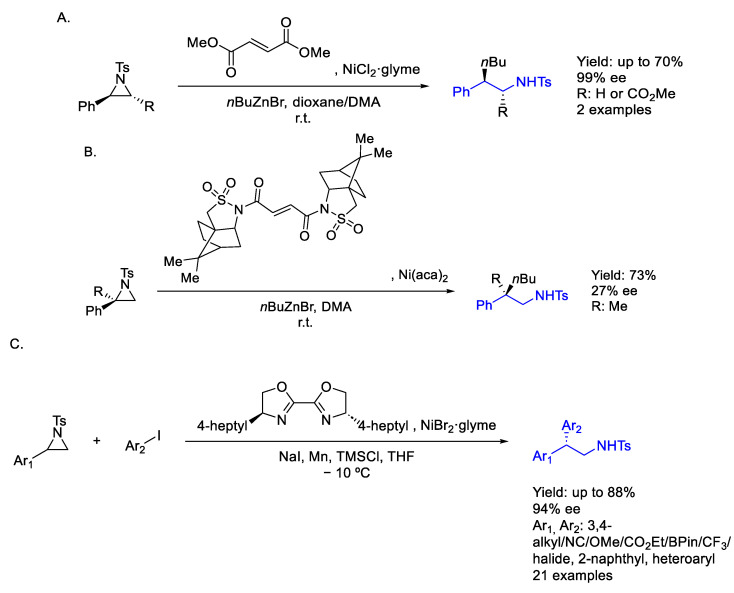
Aziridine ring-opening methodology by Doyle et al. throughout the years, synthesizing interesting chiral AEAs: (**A**) Negishi alkylation. (**B**) Chiral sultam auxiliaries ligand for Ni. (**C**) Chiral bioxazoline ligand for Ni.

Doyle et al. have been developing interesting ring-opening reactions from aziridines in order to synthesize, among others, enantiopure AEA. In 2012 [94], they achieved a nickel-catalyzed Negishi alkylation of styrenyl aziridines starting from optically active substrates, maintaining the enantiomeric excess towards the products, resulting in chiral AEAs in good yields and ee (Scheme 70A). Later, in 2015 [95], they used an organo-nickel complex as the catalyst for the ring opening for enantiopure quaternary carbon-containing aziridines, synthesizing an interesting substituted chiral AEA in moderate ee (Scheme 70B). Finally, in 2017 [96], they used a chiral organo-nickel complex to introduce chirality in the ring-opening reaction of racemic aziridines by direct asymmetric arylation, resulting in chiral diaryl ethyl amines in good yields and ee (Scheme 70C).

**Scheme 71:**
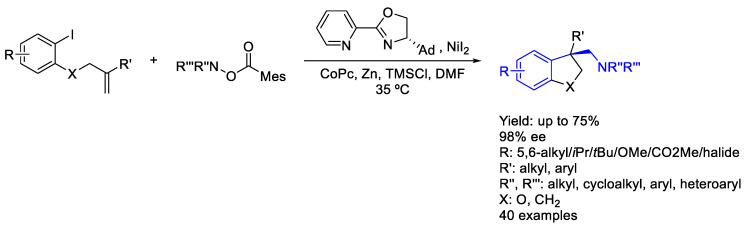
Chiral AEA based on benzofused rings by Zhu et al. in 2020.

Then, in 2020, Zhu et al. [97] developed an interesting synthesis of a chiral AEA in which the aryl group is fused either with a dihydrofurane or a cyclopentane ring (Scheme 71). Starting from diverse alkene-tethered aryl iodides and *O*-benzoyl-hydroxylamines, an asymmetric reductive 1,2-carboamination of unactivated alkenes was performed, enabled by nickel catalysis with a β-chiral diamine organo-complex, resulting in good yields and ee.

**Scheme 72:**
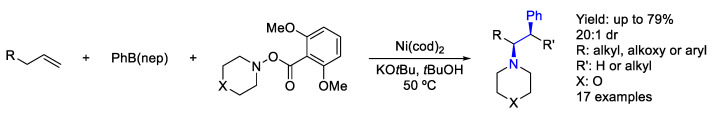
Three-component reaction towards the synthesis of chiral AEAs by Engle et al. in 2021.

Later, in 2021, an asymmetric 1,2-carboamination of alkenes was also achieved by Engle et al. [98]. Enabled by nickel catalysis, interesting chiral AEAs were synthesized through a three-component reaction from unactivated alkenes with arylboronic esters and electrophilic aminating agents (Scheme 72). This methodology relied on tailored *O*-(2,6-dimethoxybenzoyl)hydroxylamine electrophiles that suppressed competitive processes, including undesired β-hydride elimination and transesterification between the alcohol substrate and electrophile. Thus, interesting chiral AEAs were reported in good yields and dr.

**Scheme 73:**
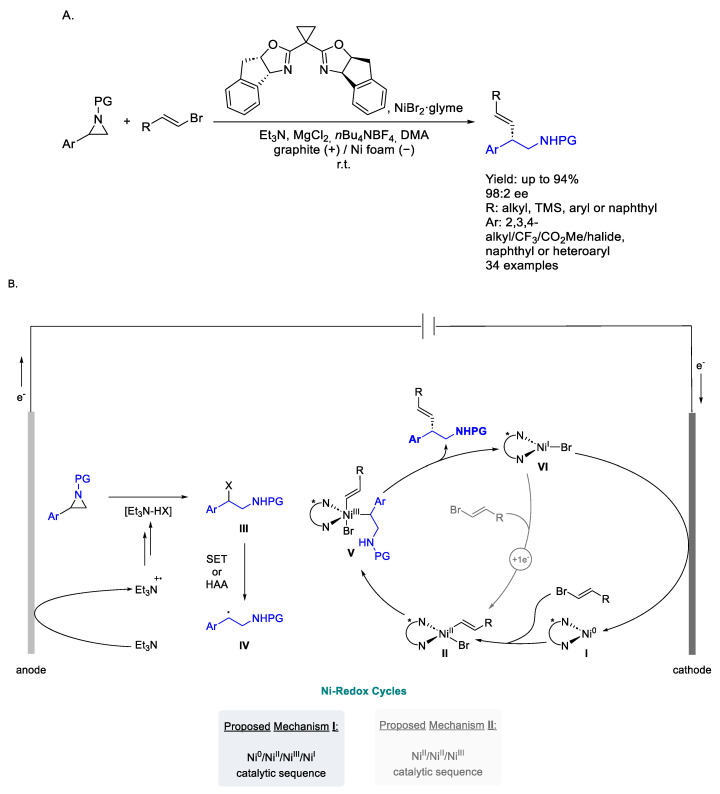
(**A**) Asymmetric synthesis of AEA by electronickel catalysis by Nevado et al. in 2023. (**B**) Proposed mechanism.

Back to asymmetric aziridine ring-opening reactions, in 2023 [99], Nevado et al. reported the use of a chiral organo-nickel complex to synthesize interesting chiral AEAs in excellent yields and ee. Starting from activated aziridines and the corresponding bromide substrates, an electrochemically driven nickel-catalyzed enantioselective reductive cross-coupling reaction was achieved, leading to a great variety of enantiopure AEAs (Scheme 73A). Interestingly, this electroreductive strategy proceeds in the absence of heterogeneous metal reductants and sacrificial anodes by employing constant current electrolysis in an undivided cell with triethylamine as a terminal reductant, as shown in the proposed catalytic cycle (Scheme 73B), making this method more atom-economical and scalable for synthetic applications.

**Scheme 74:**
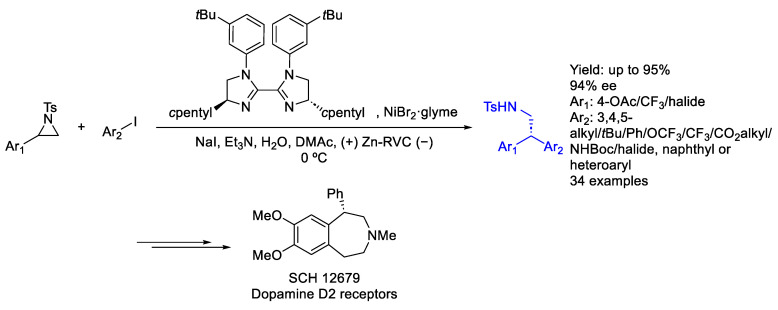
Chiral synthesis of AEAs as precursors of dopamine D2 receptor antagonist by Mei et al. in 2023.

Also in this field, in the same year, Mei et al. [100] also reported an asymmetric electrochemical reductive aziridine ring-opening reaction by the use of a chiral nickel–biimidazole catalyst complex. A reductive cross-coupling reaction from the corresponding aziridines and aryl iodide substrates led to excellent yields and ee when it came to the synthesis of a chiral AEA (Scheme 74). Similarly to Nevado et al.’s work, this reaction occurred in an undivided cell, allowing the electroreduction-mediated turnover of the nickel catalyst instead of a metal reductant-mediated turnover. Interestingly, later derivatization of the products could afford SCH 12679, a dopamine D2 receptor antagonist.

**Scheme 75:**
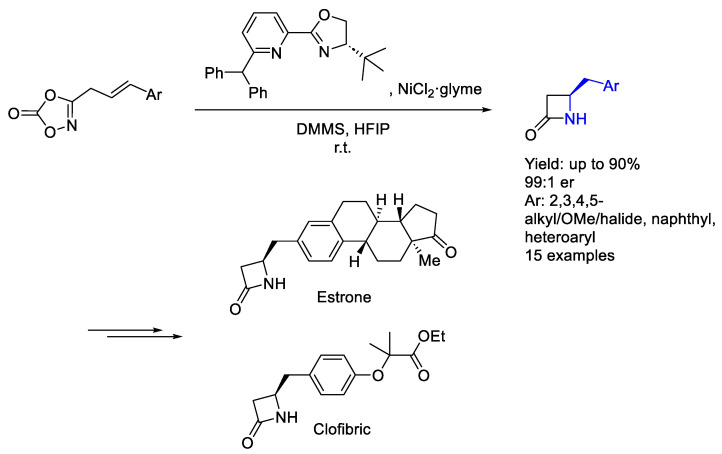
β-lactam-based chiral AEA by Chang et al. in 2023.

In the same year, Chang et al. [101] developed an asymmetric synthesis of β-lactams leading to interesting chiral AEAs in excellent yields and ee. By intramolecular hydroamination enabled by a chiral organo-nickel catalyst, this methodology afforded an enantiopure AEA via proximal C–N bond formation in which the nitrogen atom was embedded into a β-lactam ring. Later derivatization could afford interesting bioactive compounds, such as clofibric or estrone derivatives (Scheme 75).

**Scheme 76:**
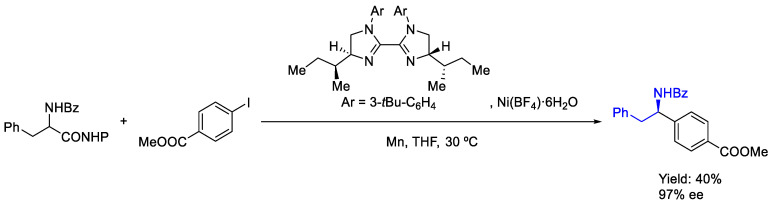
Single chiral AEA enabled by nickel catalysis by Liu et al. in 2023.

Following studies published in the same year, Liu et al. [102] used nickel catalysis to produce chiral benzylamines, synthesizing an interesting chiral AEA in the process in moderate yield and ee. An asymmetric decarboxylation of NHP esters via a reductive cross-coupling reaction was enabled by an organo-nickel complex starting from racemic benzylamine and aryl iodide substrates (Scheme 76).

**Scheme 77:**
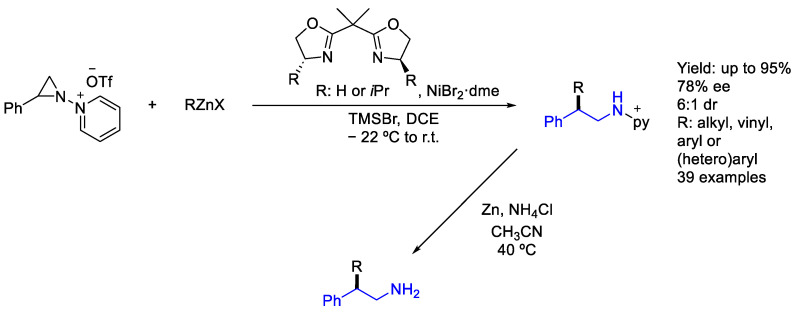
*N*-pyridinium-based AEA by Powers et al. in 2024.

Later in 2024, Powers et al. [103] reported the asymmetry ring-opening reaction of racemic *N*-pyridinium aziridines towards the synthesis of an interesting chiral AEA. Different organo-nickel complexes were used to afford *N*-pyridinium phenethylamine salts in excellent yields and ee with alkylzinc substrates, and further derivatization could afford the corresponding substituted β-phenethylamines (Scheme 77).

**Scheme 78:**
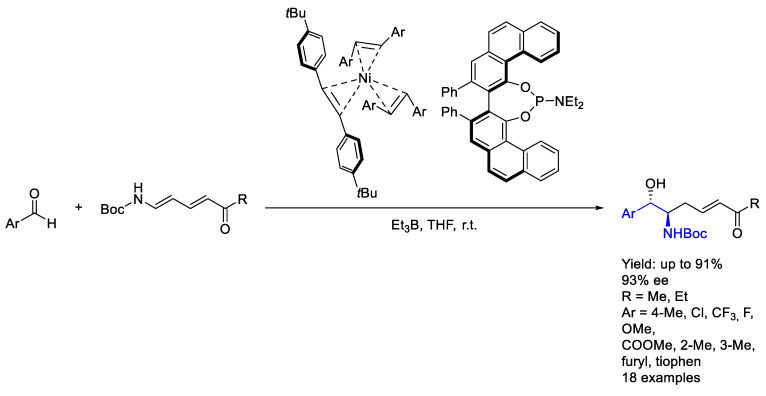
Chiral aminoalcohols as AEAs by Fürstner at al. in 2024.

Also in 2024, Fürstner et al. [104] reported a study involving the obtention of a chiral AEA by Ni catalysis. This enantioselective synthesis of *vic*-aminoalcohol derivatives by nickel-catalyzed reductive coupling of aldehydes with protected amino-pentadienoates could afford a polisubtituted asymmetric AEA in excellent yields and ee (Scheme 78). The methodology relied on reductive coupling conveniently performed with a bench-stable Ni(0) precatalyst and Et_3_B as the promoter to obtain interesting aminoalcohols.

**Scheme 79:**
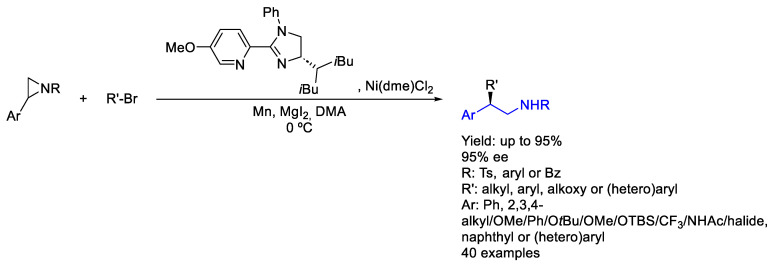
Aziridine ring-opening towards the synthesis of a chiral AEA by Liu et al. in 2024.

Liu et al. [105] reported, also in 2024, another asymmetric aziridine ring-opening reaction starting from racemic substrates. A C(sp^3^)-C(sp^3^) cross-coupling reaction from racemic *N*-sulfonyl styrenyl aziridines primary alkyl bromides was enabled by a nickel/pyridine-imidazoline complex, leading to phenethylamine derivatives with excellent yields and ee (Scheme 79).

**Scheme 80:**
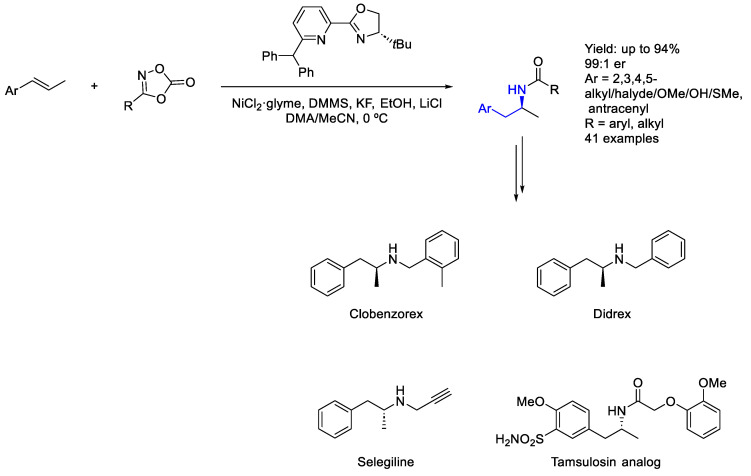
β-arylamides and their derivatization by Chang et al. in 2025.

Finally, in 2025, Chang et al. [106] achieved the asymmetric synthesis of b-arylamides by nickel-catalyzed homobenzylic hydroamidation of aryl alkenes. By employing a transposed NiH catalysis approach, this method facilitated the formation of key chiral nickel-amido intermediates, enabling asymmetric insertion into alkenes to produce the desired compounds with excellent enantioselectivity and yields. Interestingly, subsequent studies could afford corresponding bioactive compounds, such as clobenzorex, Didrex, selegiline, or tamsulosin analog (Scheme 80).

### 2.6. Iridium

**Scheme 81:**
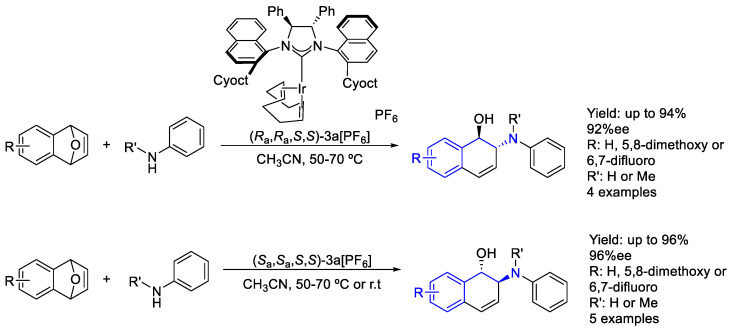
NHC-iridium catalysis towards the synthesis of asymmetric AEAs by Dorta et al. in 2020.

In 2020, Dorta et al. [107] used chiral NHC-iridium complexes to introduce chirality when synthesizing asymmetric AEAs by enantioselective intramolecular hydroaminations and ring-opening aminations. The resulting products consisted of a large series of chiral AEAs where the corresponding aryl group is always fused with a six-member ring, achieving excellent yields and ee in the process (Scheme 81).

**Scheme 82:**
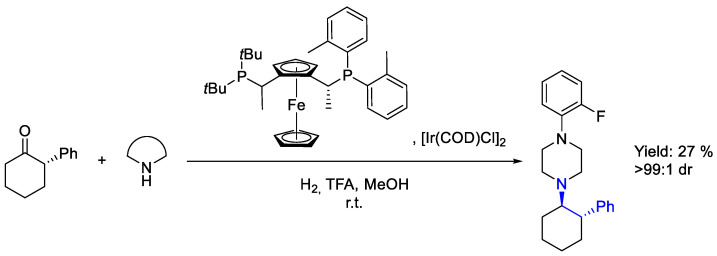
Single chiral AEA by Jouffroy et al. methodology, in 2020.

Later, in 2020, Jouffroy et al. [108] developed the synthesis of interesting polycyclic chiral products through direct reductive amination of ketones and secondary amines enabled by an in situ-generated organo-iridium complex based on a ferrocene moiety. In the process, a particular chiral polisubstituted AEA was synthesized in low yield and excellent dr (Scheme 82).

**Scheme 83:**
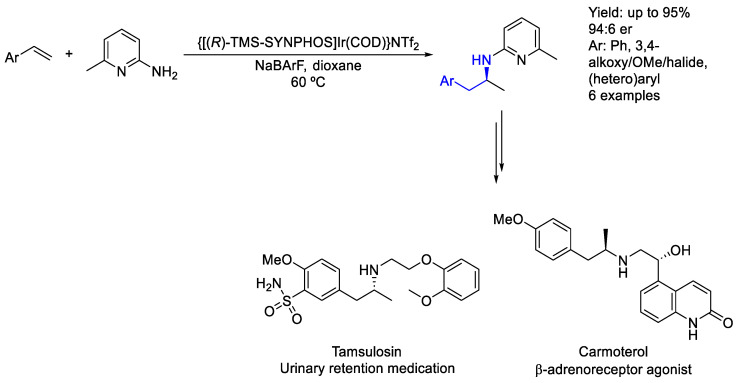
Hydroamination of unactivated alkenes towards the synthesis of a chiral AEA by Hartwig et al. in 2022.

Asymmetric hydroamination was also achieved by Hartwig et al. in 2022 [109], synthesizing an interesting chiral AEA from unactivated terminal alkenes by a chiral organo-iridium complex. This methodology used equimolar amounts of alkene and amine and, in order to achieve N-H addition, reversibility of the addition, reversible oxidation of the product amine, competing isomerization of the alkene reactant, and unfavorable replacement of sacrificial ligands in standard catalyst precursors by the chiral bisphosphine proved to be crucial in the process. Thus, asymmetric AEA synthesis was achieved in excellent yields and ee (Scheme 83).

**Scheme 84:**
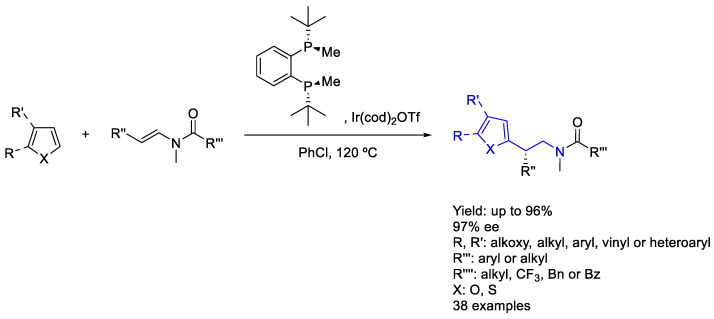
Polisubstituted chiral AEA obtained by iridium catalysis by Li et al. in 2023.

One year later, in 2023, Li et al. [110] developed a methodology in which an enantiosolective group repositioning reaction was enabled by a chiral iridium complex, synthesizing an interesting chiral AEA in which the corresponding aryl group was a hetero-five-member ring, including furan, benzofuran, and thiophene (Scheme 84). Starting from corresponding heteroarene and enamide substrates, the C–H bond at the C-2 position of the heteroarene is site-selectively cleaved and added regioselectively to the β-position of an enamide, resulting in a polisubstituted AEA in excellent yields and ee.

**Scheme 85:**
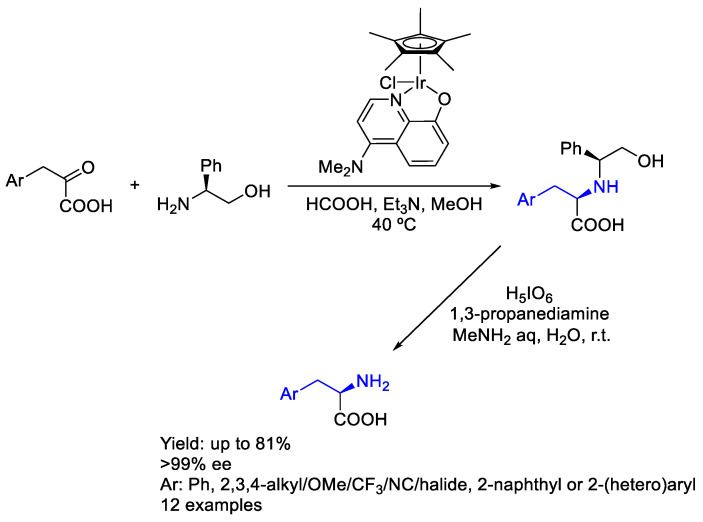
Chiral AEA as unnatural α-amino acids by Kuwata et al. in 2024.

Last year, Kuwata et al. [111] used a chiral organo-iridium complex for the asymmetric reductive amination of α-keto acids. Starting from optically active 2-phenyglycinol as the aminating agent, the resulting products could be derivatized by subsequent elimination of the hydroxyethyl moiety in order to obtain the corresponding chiral AEA as unprotected unnatural α-amino acids in good yields and ee (Scheme 85).

**Scheme 86:**
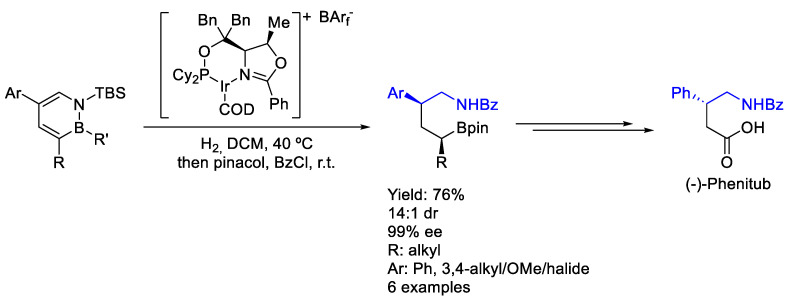
Chiral AEAs and their derivatization by Liu et al. in 2024.

Also in 2024, Liu et al. [112] synthesized interesting δ-aminoboronic esters starting from 1,2-azaborines by enantioselective hydrogenation enabled by a chiral iridium complex as the chiral introducing agent. The resulting chiral AEAs were synthesized in good yields, ee, and dr. Also, derivatization of the products led to interesting bioactive molecules, including phenibut as a chiral AEA, among others (Scheme 86).

### 2.7. Cobalt

**Scheme 87:**
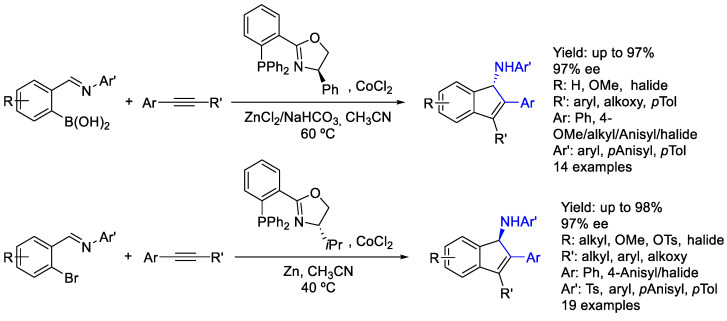
Aminoindenes as chiral AEAs by Cheng et al. in 2018.

Starting in 2018, Cheng et al. [113] synthesized 1-aminoindenes via enantioselective [3 + 2] annulation enabled by a chiral cobalt complex, resulting in highly polisubstituted chiral AEAs in excellent yields and ee (Scheme 87). The desired products could be selectively prepared in either (*R*)- or (*S*)-form by the ligand-controlled synthesis, which was initiated by the cleavage of C–B, C–Br, or C–O bonds under very mild reaction conditions.

**Scheme 88:**
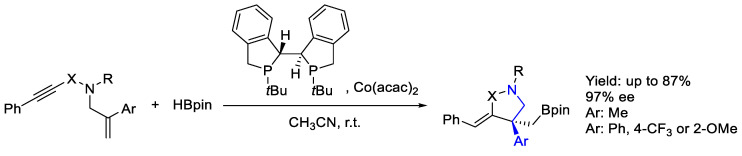
Organo-cobalt-mediated reaction towards the synthesis of a chiral AEA by Ge et al. in 2018.

Also in 2018, Ge et al. [114] reported an asymmetric synthesis of chiral boryl-functionalized γ-lactams and tetrahydro-pyrroles containing an all-carbon quaternary stereocenter by a chiral cobalt complex. The resulting asymmetric AEAs, in which the aryl moiety was present in the substrates (Scheme 88), were synthesized by a ring-closing reaction enabled by an organo-cobalt complex.

**Scheme 89:**
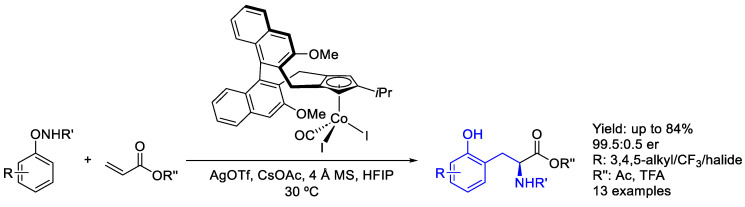
Amino acid as chiral AEA by Cramer et al. in 2020.

Then, in 2020, Cramer et al. [115] developed an enantioselective carboamination reaction by C-H activation of *N*-phenoxyamides enabled by the tailored tri-substituted chiral Cpx ligand. The resulting chiral AEAs, in which the amine group is actually an amide group, were synthesized in good yields and ee. Interestingly, products of the methodology could be derivatized in order to obtain interesting amino acid derivatives (Scheme 89).

**Scheme 90:**
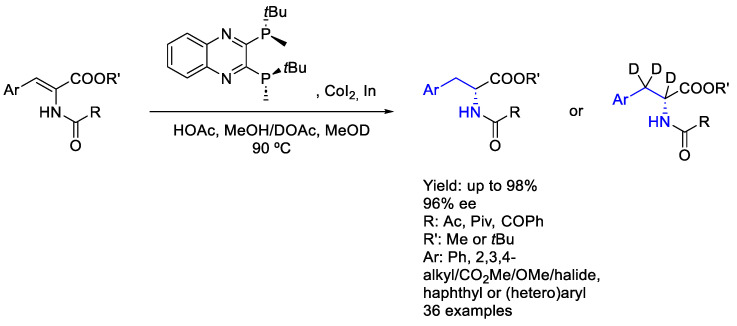
Asymmetric deuterated AEAs by Qin et al. in 2023.

Following the synthesis of amide-based AEAs, in 2023, Qin et al. [116] achieved an asymmetric deuteration of α-amidoacrylates, developing the synthesis of a chiral AEA by asymmetric hydrogenation enabled by a chiral organo-cobalt complex, resulting in excellent yields and ee using either HOAc or DOAc as hydrogenation/deuteration agent (Scheme 90). As a cheap deuterium source, the use of methanol enabled a concise synthesis of α,β-dideuterio *L*-DOPA as well as stereoselective syntheses of deuteralogs of drugs including nostocyclopeptide A2, a matrix metalloproteinase inhibitor, an anticancer agent bortezomib, nateglinide, and solriamfetol.

**Scheme 91:**
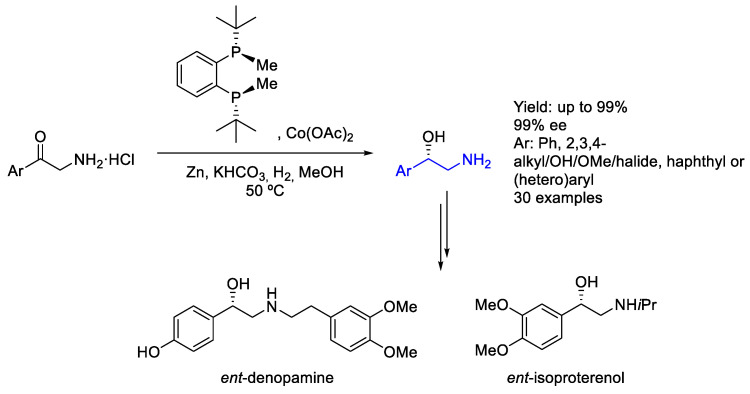
Chiral AEAs as key intermediates towards the synthesis of bioactive compounds by Zhang et al. in 2023.

In the same year, Zhang et al. [117] also achieved an asymmetric hydrogenation of ketone moieties by using a cobalt-catalyzed reaction from α-primary amino ketones. The resulting chiral AEAs were obtained in excellent yields and ee and were even used as key intermediates in the synthesis of a dopamine derivative and a potent β-adrenergic agonist, both interesting (Scheme 91).

**Scheme 92:**
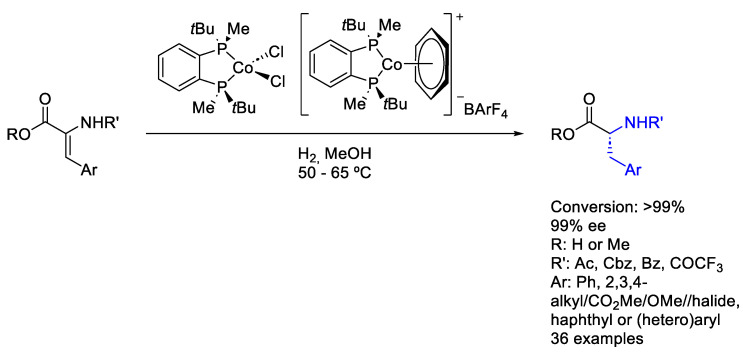
Asymmetric synthesis of an AEA enabled by cobalt catalysis by Chirik et al. in 2024.

Finally, in 2024, Chirik et al. [118] developed an asymmetric hydrogenation of aryl-containing enamides, resulting in interesting chiral AEAs in excellent yields and ee. Cobalt-catalyzed asymmetric hydrogenation was enabled by different organo-cobalt complexes containing either Co(I) or Co(II), both of them showing great performance. Thus, indazole-containing enamides relevant to the synthesis of the calcitonin gene-related peptide (CGRP) receptor antagonist, zavegepant, approved for the treatment of migraines, could be described (Scheme 92).

### 2.8. Others

#### 2.8.1. Iron

**Scheme 93:**
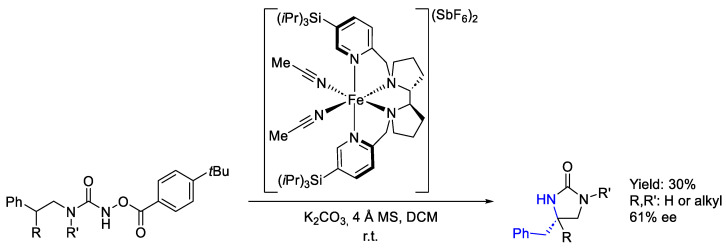
Iron-catalyzed chiral AEA by Meggers et al. in 2023.

In 2023, Meggers et al. [119] achieved the synthesis of chiral 2-imidazolidinones by using iron as the transition metal included in a chiral organic complex. By starting from branched urea-based substrates, an enantioselective ring-closing amination reaction could be undertaken, through intermediate iron nitrene species, in moderate yields and moderate ee (Scheme 93).

#### 2.8.2. Titanium

**Scheme 94:**
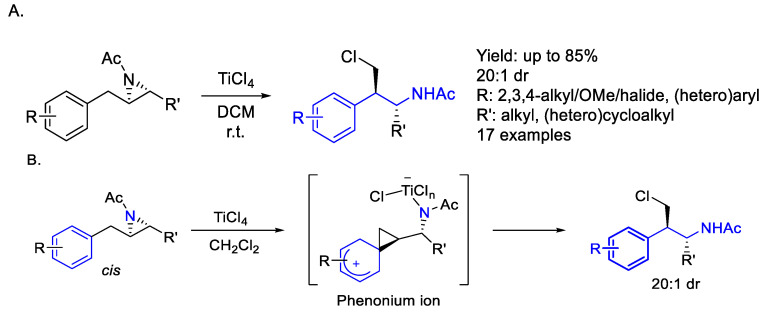
Stereoselective ring-opening reaction of aziridines (**A**) and its proposed mechanism (**B**) by Race et al. in 2021.

In 2021, Race et al. [120] developed a methodology in which a chiral AEA could be synthesized by a phenonium ion intermediate enabled by TiCl_4_. Starting from diastereopure aziridine substrates, high yields and dr were achieved in less than 10 min reaction time (Scheme 94A). Mechanistically, coordination of the aziridine to TiCl_4_ showed an energetic preference for monodentate *O*-ligation to the acyl group with subsequent phenonium ion formation. Then, chloride opening took place, resulting in diasteroseletive ring opening (Scheme 94B).

#### 2.8.3. Zinc

**Scheme 95:**
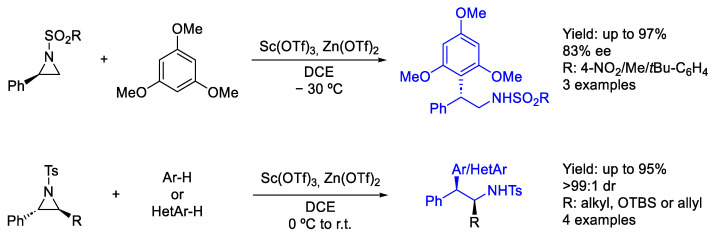
Dual catalysis enabled by Zn and Sc towards the synthesis of chiral AEAs by Ghorai et al. in 2013.

In 2013, Ghorai et al. [121] used dual catalysis of zinc and scandium Lewis acids to catalyze S_N_2-type ring opening of *N*-activated aziridines with electron-rich arenes and heteroarenes. Chiral AEAs were synthesized from the corresponding activated chiral aziridines, resulting in highly functionalized 2,2-diaryl/heteroarylethylamines in excellent yields and ee (Scheme 95).

**Scheme 96:**
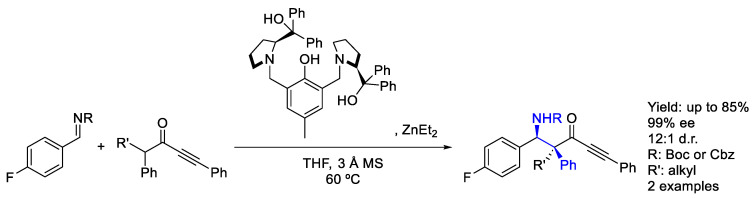
Asymmetric synthesis of AEA through Mannich reaction by Trost et al. in 2019.

Trost et al., in 2019 [122], developed an interesting synthesis of a polisubstituted chiral AEA between *N*-carbamoyl imines and α-branched ketones catalyzed by Zn-ProPhenol enabled by Mannich reactions. Key to this strategy was the introduction of unsaturation on one side of the ketone pronucleophiles, which drastically improved the reactivity and overcame the challenge of regioselectivity at the more substituted position. The resulting products with quaternary centers could be achieved in good yields and ee (Scheme 96).

## 3. Photocatalysis

The use of visible light to promote organic transformations has shown interesting and rapid advances in the last decades. When it comes to waste management and reaction efficiency, light seems to offer great advantages to be used both in the laboratory and on an industry scale [123,124]. In this sense, progress in this field has allowed us to produce ideal cheap and energy-efficient light sources available for photocatalysis. Although ultraviolet light has been used in classical photochemistry, visible light, as well as photoredox catalysis and photosensitizers, has become much easier to be used in a typical laboratory setup [125].

Since the report by MacMillan et al. in 2008 on visible-light singly occupied molecular orbital (SOMO) photoredox catalysis allowing enantioselective α-alkylations by a combination of photoredox catalysis and organocatalysis [126], photoredox catalysis using visible light has been applied to a large range of important organic transformations [127,128,129].

Photocatalysts rely either on metal complexes or on organic dyes, which can be excited by light and thus activate intermediates either by electro, hydrogen, or energy transfer. The most recent uses of different photocatalysts in combination with other techniques in organic synthesis or otherwise will be discussed.

### 3.1. Iridium

**Scheme 97:**
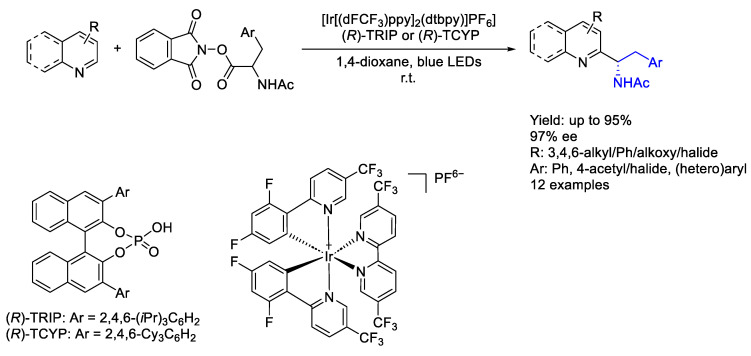
Interesting polisubstituted AEA by Phipps et al. in 2018.

In this regard, Phipps et al., in 2018 [130], reported a process for the addition of prochiral radicals from amino acid derivatives in order to synthesize chiral quinolines with asymmetric AEAs in their structure (Scheme 97). This method offered excellent enantio- and regioselective control enabled by a chiral Brønsted acid catalyst, which served both to activate the substrate and to induce asymmetry, while an iridium photocatalyst mediated in the required single electron transfer process.

**Scheme 98:**
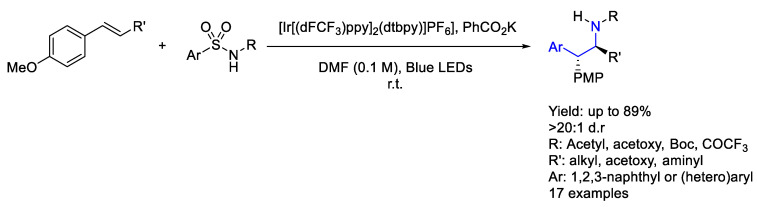
Chiral AEA by obtained via Truce–Smiles rearrangement enabled by iridium photocatalysis by Monos et al. in 2018.

Also in 2018, Monos et al. [131] reported a catalytic iridium protocol for the 1,4-aryl migration of arylsulfonylacetamides across electron-rich alkenes through Smiles–Truce rearrangement. This redox-neutral, visible light-driven, single-electron alkene oxidation method achieved complete anti-Markovnikov regioselectivity and excellent diastereoselectivity, enabling the synthesis of a chiral AEA from commercially available aryl sulfonamides and unactivated alkenes under mild conditions in excellent ee and yields (Scheme 98).

**Scheme 99:**
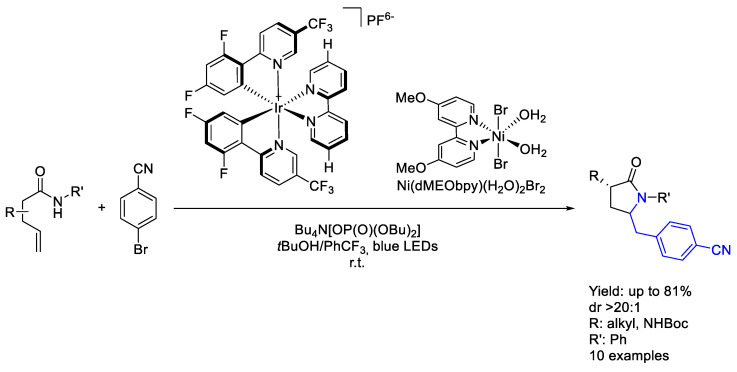
Pyrrolidinone-based chiral AEA by Molander et al. in 2019.

In 2019, Molander et al. [132] achieved a rapid and highly diastereoselective amidoarylation of unactivated olefins by a cascade process by merging, for the first time, photoredox proton-coupled electron transfer (PCET) with nickel catalysis. This novel process started with *N*-radical formation by PCET, enabled by a photoexcited iridium photocatalyst and a base, followed by an intramolecular ring-closing reaction and radical addition to nickel complex. Then, oxidative addition and reductive elimination afforded the corresponding chiral AEA in which the nitrogen atom was embedded into a pyrrolidinone ring, in good yield and dr (Scheme 99).

**Scheme 100:**
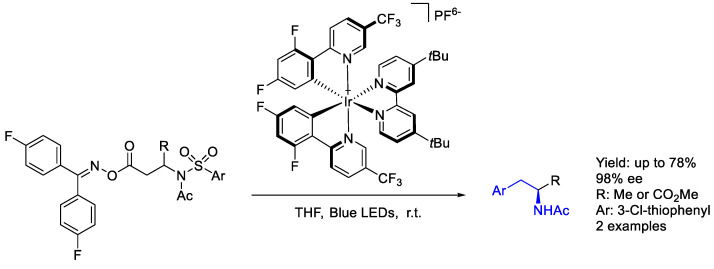
Chiral AEA obtained through Truce–Smiles rearrangement by Greaney et al. in 2020.

In 2020, Greaney et al. [133] developed a new powerful decarboxylative, desulfonylative Truce–Smiles rearrangement enabled by visible light energy transfer catalysis. The reaction used starting materials derived from commercially available β-amino acids, giving extensive control over the aryl species, ethyl chain substitution, and amino protection for the synthesis of a new chiral AEA in good yields and ee (Scheme 100). This methodology relied on imine activation by energy transfer from the excited iridium catalyst, followed by homolytic N–O bond cleavage and hydrogen atom transfer by the corresponding solvent.

**Scheme 101:**
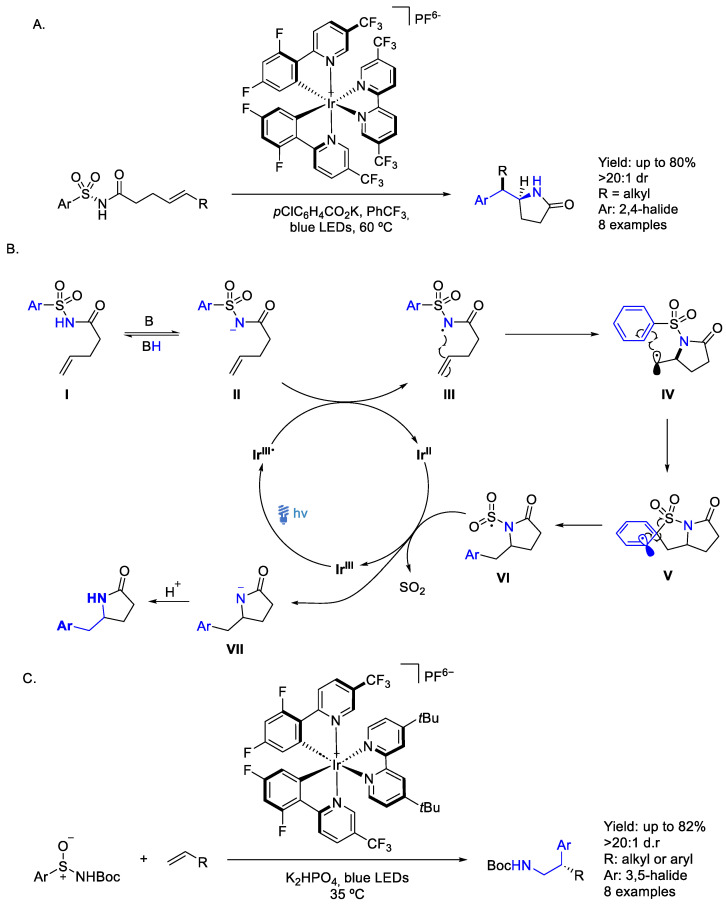
Chiral AEA from high-oxidation-state sulfur present in sulfunamide moieties (**A**), its proposed catalytic cycle (**B**), and from low-oxidation-state sulfur present in sulfinimide moieties (**C**).

Later, in 2022, Stephenson et al. [134] developed an intramolecular aromatic *ipso* substitution of a heteroatom leaving group by a carbon nucleophile with sulfonamides as bifunctional aryl and amine sources. This Smiles–Truce rearrangement was enabled by single electron transfer from the excited iridium catalyst to the corresponding sulfonamide substrate. Interestingly, it could only afford the desired chiral AEA when *O*-substituents were present in the aryl moiety, blocking dearomatization, giving the corresponding lactams in good yields and dr (Scheme 101A). Two years later, in 2024 [135], following their studies, they transferred the Smiles–Truce rearrangement to the aminoarylation of unactivated alkenes by lowering the oxidation state of the sulfur atom from S(VI) to S(IV), starting from the corresponding sulfinamides (Scheme 101B). The resulting change to the sulfur atom’s molecular geometry contracted the C–S–N bond angle, thereby favoring the *ipso* cyclization step of the aryl migration. Using a weakly oxidizing photoredox catalyst, a sulfinamidyl radical was generated under mild conditions by single electron transfer from the iridium photocatalyst and was added to an alkene to form a new C–N bond, followed by a desulfinylative Smiles–Truce rearrangement to form a new C–C bond. Thus, interesting chiral AEAs were synthesized in good yields and dr (Scheme 101C).

**Scheme 102:**
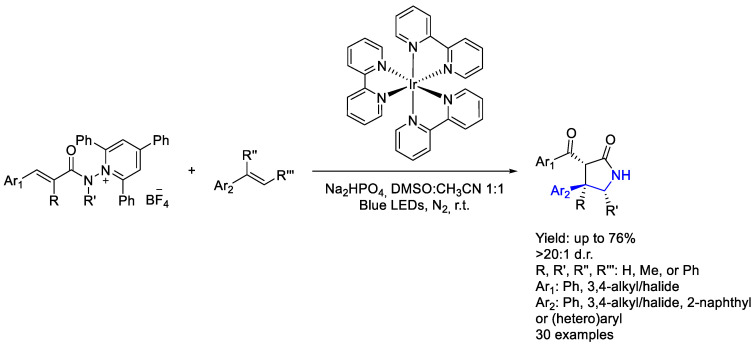
γ-lactam synthesis as chiral AEA by Xia et al. in 2022.

In 2022, Xia et al. [136] demonstrated a methodology for a photoinitiated deaminative [3 + 2] annulation reaction of *N*-aminopyridinium salts with alkenes for the synthesis of functionalized γ-lactams enabled by a chiral iridium photocatalyst. The corresponding synthetized chiral polisubstituted AEAs had their nitrogen atoms embedded into a γ-lactam ring (Scheme 102). The mechanism relied on energy transfer from the iridium photocatalyst to the corresponding salts to enable homolytic N-N cleavage, followed by sterenyl addition, an intramolecular ring-closing process, and subsequent quenching with DMSO.

**Scheme 103:**
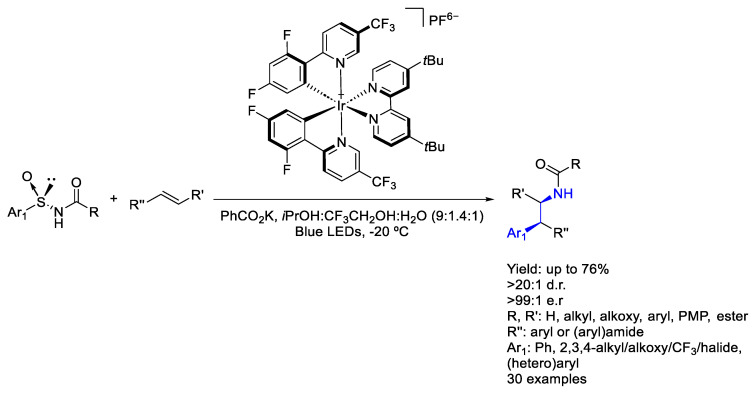
β,β-diarylethylamines as asymmetric AEAs by Nevado et al. in 2023.

Nevado et al., in 2023 [137], described enantioenriched arylsulfinylamides as all-in-one reagents for the efficient asymmetric, intermolecular aminoarylation of alkenes. Under mild photoredox conditions, nitrogen addition of the arylsulfinylamide onto the double bond, followed by 1,4-translocation of the aromatic ring, afforded, in a single operation, production of the corresponding aminoarylation adducts in an enantiomerically enriched form (Scheme 103). Mechanistic investigations revealed the likelihood of multiple reaction pathways operating in these transformations. In the case of electron-rich styrenes, the proposed mechanism was based on radical cation formation by single-electron oxidation. In contrast, the single-electron oxidation of the deprotonated arylsulfinylamide by the excited iridium photocatalyst to form an *N*-centred radical seemed to occur in the case of poorly oxidizable olefins. This methodology could afford interesting optically pure β,β-diarylethylamines in good yields and ee.

**Scheme 104:**
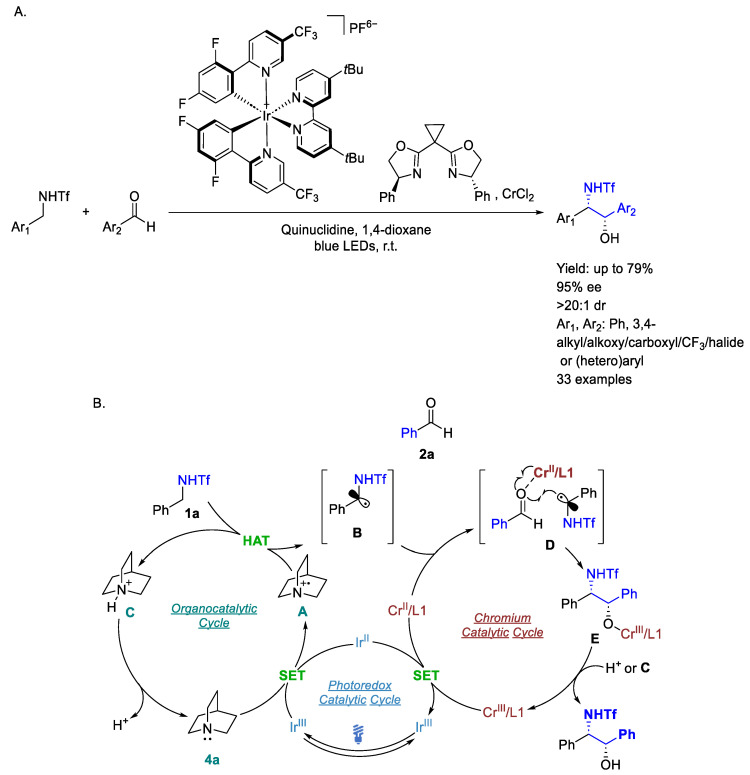
Iridium-catalyzed synthesis of chiral AEA by Wang et al. in 2024 (**A**) and its proposed catalytic cycle (**B**).

Wang et al., in 2024 [138], presented a novel synergistic triple-catalysis approach for the asymmetric α-C–H addition of readily available *N*-sulfonyl amines to aldehydes under mild conditions. This method allowed the efficient synthesis of a diverse variety of valuable β-amino alcohols bearing vicinal stereocenters, affording an interesting chiral AEA in good yield and ee (Scheme 104A). An iridium photocatalyst enables quinuclidine activation by the single electron transfer process, which, by hydrogen atom transfer, activated the corresponding benzylamine substrate, proceeding towards its addition to the aldehyde–chrome complex, affording the aforementioned products (Scheme 104B).

### 3.2. Ruthenium

**Scheme 105:**
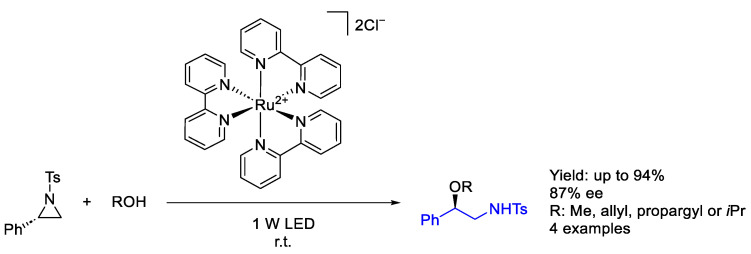
1,2-bifunctional AEA by Xia et al. in 2014.

In 2014, Xia et al. [139] developed a mild and efficient procedure for the regioselective ring-opening nucleophilic addition reactions of aziridines via visible light photoredox catalysis, which provided practical synthetic access to 1,2-bifunctional compounds, synthesizing chiral AEAs in the process. Starting from the corresponding optically active aziridine, the mechanism relied on single electron transfer from the excited ruthenium photocatalyst towards the substrates, followed by nucleophilic ring opening and subsequent hydrogen atom transfer from the solvent, affording the desired products in excellent yields and ee (Scheme 105).

**Scheme 106:**
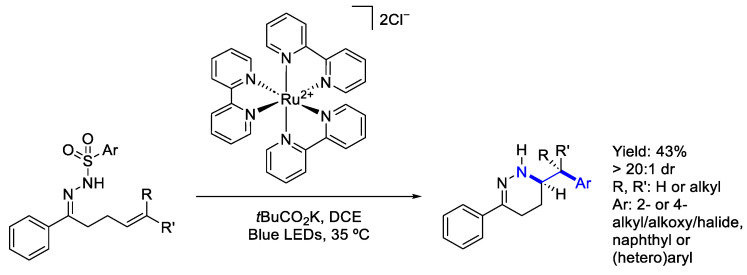
Polisubstituted chiral AEA based on 1,4,5,6-tetrahydropyridazines by Liu et al. in 2021.

Liu et al., in 2021 [140], developed a visible light-mediated approach for synthesizing 1,4,5,6-tetrahydropyridazines with arylethylamine motifs using *N*-radical cyclization and Smiles–Truce aryl transfer. This cascade reaction, enabled by a photoredox catalyst and a base, operated under mild, redox-neutral conditions and demonstrated excellent functional group compatibility and diastereoselectivity. Additionally, it enabled the construction of synthetically challenging all-carbon quaternary centers through alkene aminoarylation. Single electron transfer from the excited ruthenium photocatalyst towards the corresponding substrates followed by an intramolecular ring-closing reaction and the desulfonation process afforded the aforementioned chiral AEA in moderate yields and dr (Scheme 106).

### 3.3. Nickel

**Scheme 107:**
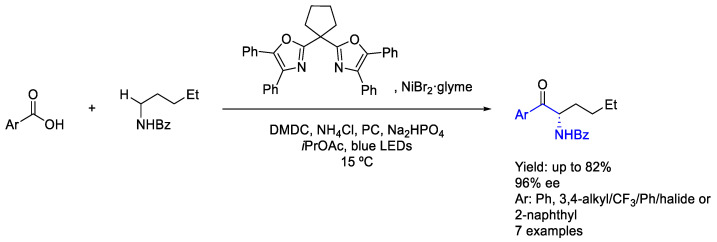
Asymmetric synthesis of an AEA enabled by nickel catalysis by Huo et al. in 2020.

Subsequently, Huo et al., in 2020 [141], achieved a direct enantioselective acylation of α-amino C(sp^3^)–H bonds with carboxylic acids via the combination of a transition metal and photoredox catalysis. This straightforward protocol enabled cross-coupling of a wide range of carboxylic acids, with readily available *N*-alkyl benzamides to produce chiral AEAs in high enantioselectivities under mild conditions. A photoexcited nickel catalyst enabled Br radical formation, which prompted hydrogen atom transfer with the corresponding substrates to form radical intermediates, followed by oxidative addition of the chiral nickel organo-catalyst and reductive elimination to afford the desired products (Scheme 107).

**Scheme 108:**
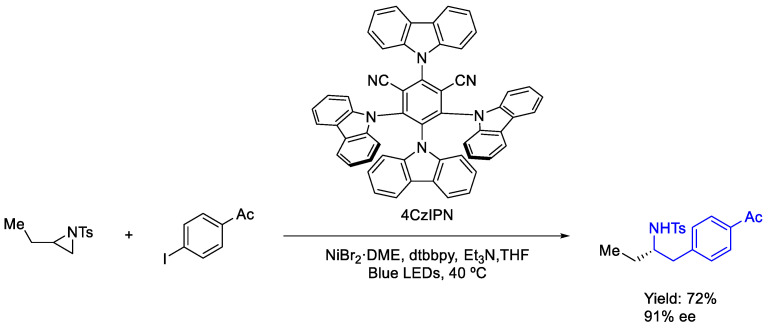
*N*-tosyl-based AEA by Doyle in 2020.

Doyle et al., in 2020 [142], reported a photoassisted nickel-catalyzed reductive cross-coupling between tosyl-protected alkyl aziridines and commercially available (hetero)aryl iodides. 4CzIPN as the photocatalyst enabled single electron transfer from the aminoiodine intermediate towards aziridine substrates, followed by oxidative addition and reductive elimination of the corresponding chiral organo-nickel complex, affording an interesting chiral AEA, when starting from the optically active substrate, maintaining the stereochemistry in good yield (Scheme 108).

**Scheme 109:**
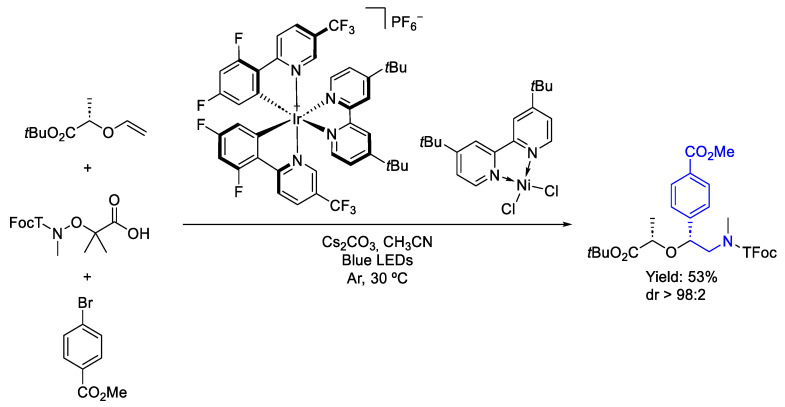
Chiral AEA incorporating ether moiety by Studer et al. in 2021.

Studer et al., in 2021 [143], reported a three-component stereoselective 1,2-aminoarylation of electron-rich alkenes through synergistic photoredox and nickel catalysis. Anti-Markovnikov addition of the amidyl radical to the alkene and nickel-mediated radical/transition metal crossover led to the corresponding 1,2-aminoarylation product, offering a route for the preparation of enantiopure α-arylated β-amino alcohols. In this process, interesting chiral AEAs were synthesized in moderate yields and excellent dr (Scheme 109). The mechanism involved single electron transfer from an excited organocatalyst towards the carboxylic acid substrates; sequential fragmentation of CO_2_ and acetone which generates the electrophilic N-radical; followed by addition to the corresponding alkene. Then, radical trapping by the Ni-complex followed by reductive elimination afforded the desired products.

### 3.4. Copper

**Scheme 110:**
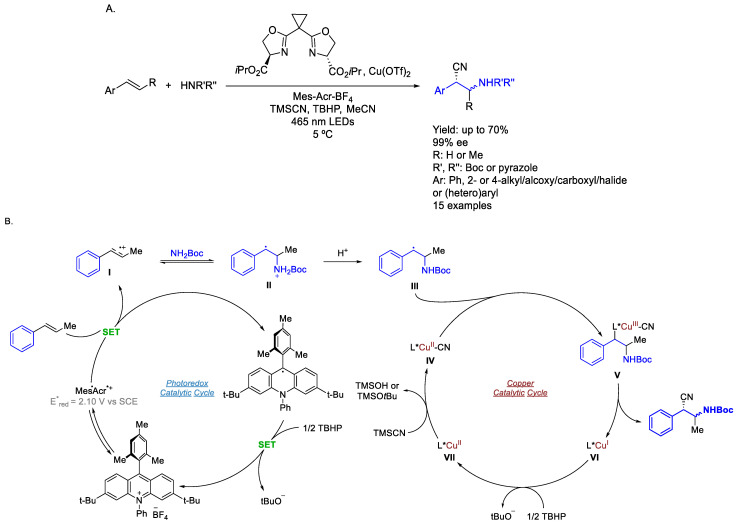
α-Ciano AEA by Nicewicz et al. in 2023 (**A**) and its proposed catalytic cycle (**B**).

Nicewicz et al., in 2023 [144], described for the first time an enantioselective difunctionalization of olefins via a cation radical intermediate utilizing an acridinium photooxidant in conjunction with copper catalysis. The transformation could be rendered asymmetric by using a serine-derived bisoxazoline ligand. The wide array of nucleophiles in this three-component coupling allowed the synthesis of an interesting chiral α-cyano AEA in good yields and great dr and ee (Scheme 110A). The scope of amines for the aminocyanation reaction was greatly expanded by undergoing a cation radical intermediate as opposed to previous *N*-centered radical-initiated aminocyanations, as shown in the proposed mechanistic cycle (Scheme 110B).

**Scheme 111:**
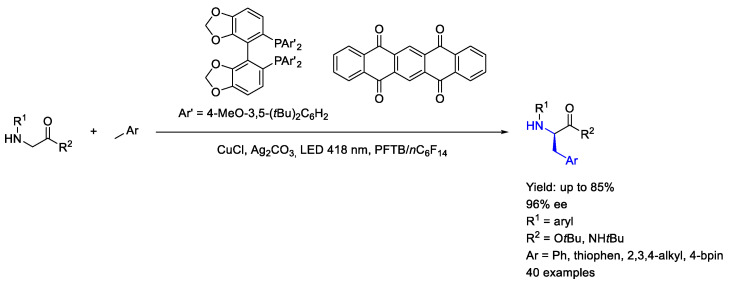
Copper catalysis towards the synthesis of interesting AEAs by Gong et al. in 2025.

In 2025, Gong et al. [145] developed a methodology towards the synthesis of β,β-aminoacyl AEA (Scheme 111). Starting from glycine derivatives, this photoinduced regiodivergent and enantioselective cross-coupling relied on a catalytic system that integrated photoinduced hydrogen atom transfer (HAT) and chiral copper catalysis. This strategy facilitated regiodivergent and enantioselective cross-couplings between *N*-aryl glycine ester/amide derivatives and abundant hydrocarbon feedstocks through strong C(sp^3^)-H bond activation, obtaining interesting AEAs in good yields and ee.

### 3.5. Wolframium

**Scheme 112:**
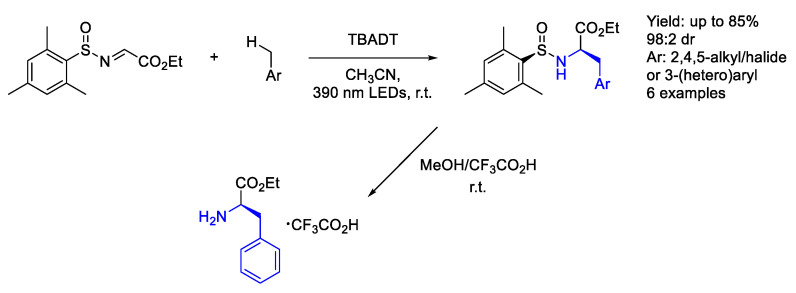
Wolframium-catalyzed asymmetric synthesis of an AEA by Masson et al. in 2024.

Masson et al., in 2024 [146], described a sustainable and efficient photocatalytic method for the stereoselective radical alkylation of chiral sulfinyl imines. By employing readily available non-prefunctionalized radical precursors and cost-effective TBADT (Tetrabutylammonium decatungstate) as a direct HAT photocatalyst, they successfully obtained diverse chiral AEAs with high yields and excellent diastereoselectivity under mild conditions (Scheme 112). Excited TBADT underwent a hydrogen atom transfer towards the corresponding methylaryl substrates, followed by sulfinyl imine addition and subsequent back-hydrogen atom transfer from the photocatalyst. Thus, the chiral AEA was synthetized with both good yield and ee.

### 3.6. Iron

**Scheme 113:**
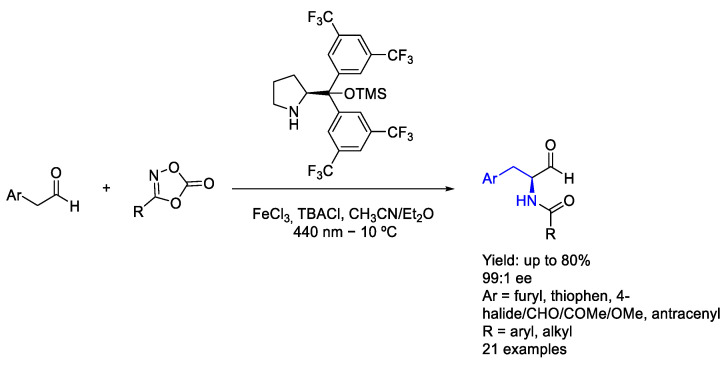
Chiral 2-arylethylacetamides by Chang et al. in 2024.

Also in 2024, Chang et al. [147] reported a visible-light-promoted enantioselective a-amidation of aldehydes by organo-iron catalysis towards the synthesis of chiral 2-arylethylacetamides (Scheme 113). Interestingly, mechanistic studies revealed that in situ-generated [Fe(II)Cl_3_^−^] via visible-light-promoted LMCT effectively activated dioxazolones, affording an iron-acylnitrenoid radical that was subsequently inserted into chiral enamine intermediates. Thus, good yields and ee were achieved.

## 4. Conclusions

Because of their very well-known bioactive properties, 2-arylethylamine motif compounds are of great interest in both synthetic and medicinal chemistry. As small nitrogen-containing compounds, they are able to pass the blood–brain barrier; thus, they are used in treatments related to Central Neural System. The human body is chiral and, thus, chirality introduction into synthetic methodologies is a mandatory approach in the pharmaceutical industry at present. This asymmetric synthesis can present several challenges when faced in a laboratory. Herein, we present the most recent advances in asymmetric synthesis of 2-arylethylamines using transition metal catalysis as the main tool to introduce chirality in either C-1, C-2, or in both carbons of the aforementioned motif. The main methodology when it comes to chiral synthesis is presented and discussed, considering conditions, challenges, yields, and ee/dr. Also, when considered, interesting and novel catalytic cycles are presented and discussed.

Conventional metal catalysis supported with a chiral organo complex is the main core of the review, organized by metal abundancy in the literature of recent years. While copper and palladium-based catalysis are the richest when it comes to different substrates and targets, rhodium and cobalt have mainly been used for asymmetric hydrogenation or hydroamination of different substrates. On the other hand, less abundant metals such as ruthenium, iridium, or nickel are not focused on one type of methodology, also considering that iridium is mainly used in photocatalysis. Photocatalysis is presented as separate section covering the main advances in recent years, showing that iridium is the most abundant transition metal used towards the synthesis of chiral AEAs.

Derivatization of the aforementioned compounds has proved to be very important in the pharmaceutical industry, as shown in the presented review.

## Data Availability

No new data were created or analyzed in this study. Data sharing is not applicable to this article.
